# Design of 8-mer peptides that block *Clostridioides difficile* toxin A in intestinal cells

**DOI:** 10.1038/s42003-023-05242-x

**Published:** 2023-08-26

**Authors:** Sudeep Sarma, Carly M. Catella, Ellyce T. San Pedro, Xingqing Xiao, Deniz Durmusoglu, Stefano Menegatti, Nathan Crook, Scott T. Magness, Carol K. Hall

**Affiliations:** 1 Department of Chemical Engineering, North Carolina State University, Raleigh, NC 27695-7905 USA; 2 Department of Medicine, University of North Carolina at Chapel Hill, Chapel Hill, NC 27514 USA; 3 Biomanufacturing Training and Education Center (BTEC), North Carolina State University, Raleigh, NC 27695 USA

**Keywords:** Computational biophysics, Diarrhoea

## Abstract

Infections by *Clostridioides difficile*, a bacterium that targets the large intestine (colon), impact a large number of people worldwide. Bacterial colonization is mediated by two exotoxins: toxins A and B. Short peptides that can be delivered to the gut and inhibit the biocatalytic activity of these toxins represent a promising therapeutic strategy to prevent and treat *C. diff*. infection. We describe an approach that combines a *Pep*tide *B*inding *D*esign (PepBD) algorithm, molecular-level simulations, a rapid screening assay to evaluate peptide:toxin binding, a primary human cell-based assay, and surface plasmon resonance (SPR) measurements to develop peptide inhibitors that block Toxin A in colon epithelial cells. One peptide, SA1, is found to block TcdA toxicity in primary-derived human colon (large intestinal) epithelial cells. SA1 binds TcdA with a K_D_ of 56.1 ± 29.8 nM as measured by surface plasmon resonance (SPR).

## Abstract (web summary)

A computational design and experimental screening approach leads to the identification of peptide inhibitors that block Toxin A in colon epithelial cells.

## Introduction

*Clostridioides difficile (C. diff*.) is a Gram-Positive, spore-forming bacterium that infects the intestinal tract of humans and animals. In the last decade, *C. diff*. infection has been the leading cause of diarrhea and inflammation of the colon in North America and in Europe^1^. In many cases, *C. difficile* infection is the consequence of a microbial imbalance caused by overtreatment with antibiotics such as penicillin, carbapenem, and fluoroquinolone^2,3^. These disrupt the gut microbiome, allowing the germination of *C. diff*. spores and leading to the proliferation of bacteria and the subsequent release of virulent toxins. In 2017, more than 200 K people were infected with *C. diff*. resulting in 12,800 deaths in the United States alone^4,5^. Most of the infections are associated with in-patient care, and more than 80% of the deaths occur in people above 65 years in age^6^. The colonic epithelium is the primary site of infection as the epithelial cells that line the gut wall are highly sensitive to the effects of *C. diff* toxins and *C. diff* preferentially colonizes the colon^7,8^.

**Fig. 1:**
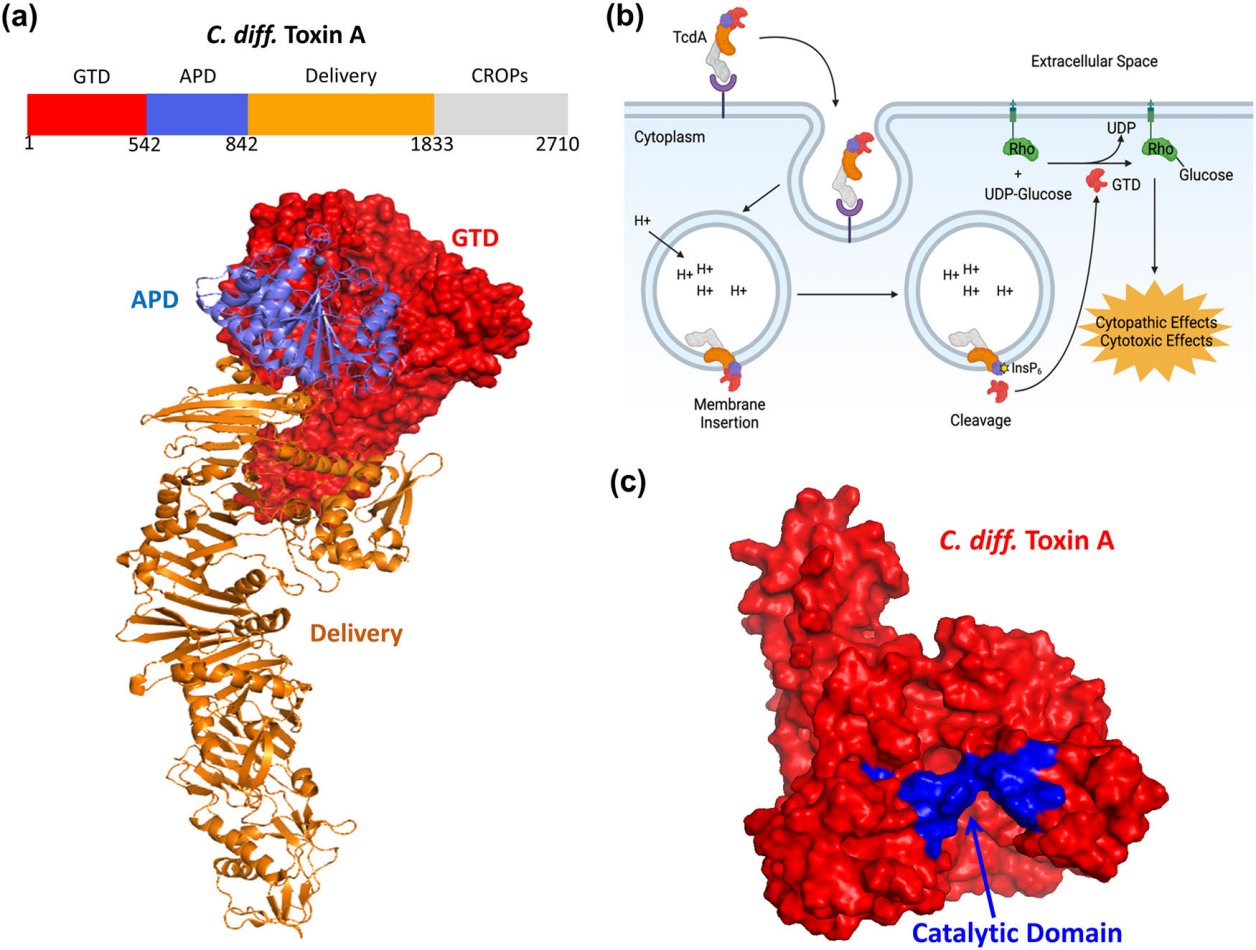
*Clostridoides difficile* Toxin A pathogenecity. **a** The crystal structure of Toxin A with the glucosyltransferase domain (GTD, red), autoprotease domain (APD, blue) and delivery domain (orange) (PDB ID: 4R04) is illustrated. **b** Schematic of the TcdA induced toxicity in human epithelial cells. **c** The catalytic site of TcdA (shown in blue) in the GTD (red) plays an important role in inducing *C. diff*. infection.

The pathogenicity of *C. diff*. derives primarily from two major toxins: Toxin A (TcdA) and Toxin B (TcdB)^9,10^. *C. diff*. adheres to the gut wall using its surface layer proteins and produces two large Rho-glucosylating toxins, TcdA and B, that share ~63% sequence homology^11,12^. These toxins comprise four domains: glucosyltransferase domain (GTD), autoprotease domain (APD), delivery domain and the combined repetitive oligopeptides domain (CROP) (Fig. 1a). The *C. diff*. toxins act via a four-step intracellular mechanism (Fig. 1b) : (1) The CROP domain, which is at the C-terminus of the toxins, binds to carbohydrate molecules and proteins on the surface of colonic epithelial cells^13–15^; (2) the delivery domain helps translocate the toxin into the cytosol of the target cells; (3) the APD cleaves the GTD from the rest of the toxin; and (4) the GTD utilizes uridine diphosphate glucose (UDP-glucose) to glucosylate Rho-family GTPases that are present in intestinal epithelial cells. The glucosylation of these Rho-family GTPases disrupts transcription, cell cycle progression, apoptosis, and cytoskeleton regulation, leading to cytopathic and cytotoxic effects^16–19^.

Multiple therapeutic approaches have been developed to treat *C. diff*. infection. The standard practice is treatment with antibiotics (metronidazole and vancomycin), but in 20% of cases infection reoccurs^20^. Exposure to these antibiotics alters the microbial community in the gut, facilitating colonization by *C. diff*^21^. Merck introduced a monoclonal antibody, Bezlotoxumab, (marketed as Zinplava) that targets *C. diff* toxin B. While the rate of recurrent infection among patients receiving Bezlotoxumab was substantially lower than for antibiotic-treated cohorts, the high cost of a single dose (~$4 K) and its intravenous infusion are burdensome^22,23^. Another *C. diff*. treatment is Fecal Microbiota Transplant (FMT), an investigational treatment not yet approved by the FDA^24^. The methods of FMT administration and optimal dosing strategies still vary from case to case. Additionally, FMT carries the risk of transmitting infectious diseases and antibiotic-resistant bacteria^25^.

Short peptides are promising candidates for the prevention and treatment of *C. diff*. infection as they are cost effective and can be specific in action. Hence, the goal of this study is to identify peptide inhibitors that bind the catalytic domain of *C. diff*. Toxin A GTD by combining computational design, molecular-level simulations, and experimental refinement. To do this we employ PepBD, a computational *pep*tide *b*inding *d*esign (PepBD) algorithm developed in our group, which performs high-throughput screening of peptide binders to biomolecular targets^26–29^, e.g., proteins and RNA. The PepBD algorithm has been used successfully in the past to design 15-mer transfer RNA^Lys3^-binding peptides^30^, peptides that recognize cardiac troponin I^31^ and neuropeptide Y^32^, peptide ligands that bind to the Fc and Fab domains of immunoglobulin G^33,34^ and peptides that bind to the Receptor Binding Domain of the SARS-CoV-2 spike-protein^35^. In an effort to rank and appraise the computationally suggested peptides, a microfluidic bead-based platform was used to rapidly identify peptides that exhibit the desired binding characteristics. This system uses fluorescence imaging and automated image analysis to measure the propensity for both on-target and off-target binding and has previously been applied to identify peptides that bind specifically to Cas9, VCAM-1, and IgG Fab fragments^36–38^. The efficacy of the peptides is tested via a trans-epithelial electrical resistance (TEER) assay on monolayers of the human gut epithelial culture model and via surface plasmon resonance measurements.

The starting point for the work described in this paper is our previously reported computationally identified 10-mer peptide, “NPA”, that binds to TcdA GTD^39^. Our choice of protein target is TcdA GTD as it builds upon the research conducted in the Feig lab at Wayne State University and addresses the unmet need for efficacious therapeutics targeting TcdA^40^. In their study, they employed phage display to identify short peptides that demonstrate binding affinity to TcdA GTD. This peptide neutralizes TcdA in differentiated small intestinal absorptive cells (SI) but has no effect on differentiated colon absorptive cells. While the mechanisms for this observation are unknown, a possible explanation is that proteases present on the brush border of SI cells cleave the 10-mer peptides into shorter, more-active forms that neutralize the toxins in the SI cells. Since the colon epithelial cells do not appreciably express proteases^41^, the 10-mer peptides are less likely to be cleaved in colon cells than in SI cells, and hence cannot neutralize the toxins in the colon cells.

In this work, we computationally design engineered variants of the NPA peptide shorter than 10 amino acids with the goal of identifying effective inhibitors of *C. diff*. TcdA. We begin by performing molecular dynamics simulations of fragments of NPA to see which of them can bind to the catalytic site of TcdA GTD. The simulation results predict that 8-mer peptide candidates are optimum. Hence, we apply the PepBD algorithm to design 8-mer peptide sequences with 8-mer NPA as the “reference peptide”. Explicit solvent atomistic MD simulations and binding free energy calculations are carried out to evaluate the binding of the *in-silico-*suggested peptides to the TcdA GTD in solution. The peptides are rapidly screened for TcdA binding and TcdA GTD binding through an in-house bead-based peptide display system to eliminate weak peptide inhibitors. The efficacy of the peptides that make it through the bead-based peptide display assay are tested using a trans-epithelial electrical resistance (TEER) assay on monolayers of the human gut epithelial culture model. While conventional cellular toxicity assays for *C. diff* toxins use non-physiologically relevant colon cancer cells or transformed kidney epithelial cells, here we use primary human gut epithelial stem cells from the large intestine (descending colon) that are differentiated in the main lineage (absorptive) of the gut lining. The experimental binding affinity of the top performing peptide to TcdA is characterized using surface plasmon resonance (SPR).

Highlights of our results are as follows. Seven candidate peptide inhibitors (SA1-SA7) were identified using our PepBD algorithm coupled with molecular level simulations. Based on the bead-based peptide display screen for TcdA GTD binding, four peptides (SA1-SA4) were selected for further in vitro assessment. SA1 was the only peptide that demonstrated neutralization properties of TcdA in the colon. The dissociation constant, K_D_, of SA1 to TcdA measured by SPR is 56.1 ± 29.8 nM. These findings suggest that peptide SA1 might be an effective therapeutic drug to treat *C. diff*. infection.

## Results

### Determining the optimal sequence length and initial peptide sequence for designing peptide inhibitors of TcdA GTD catalytic domain

**Fig. 2:**
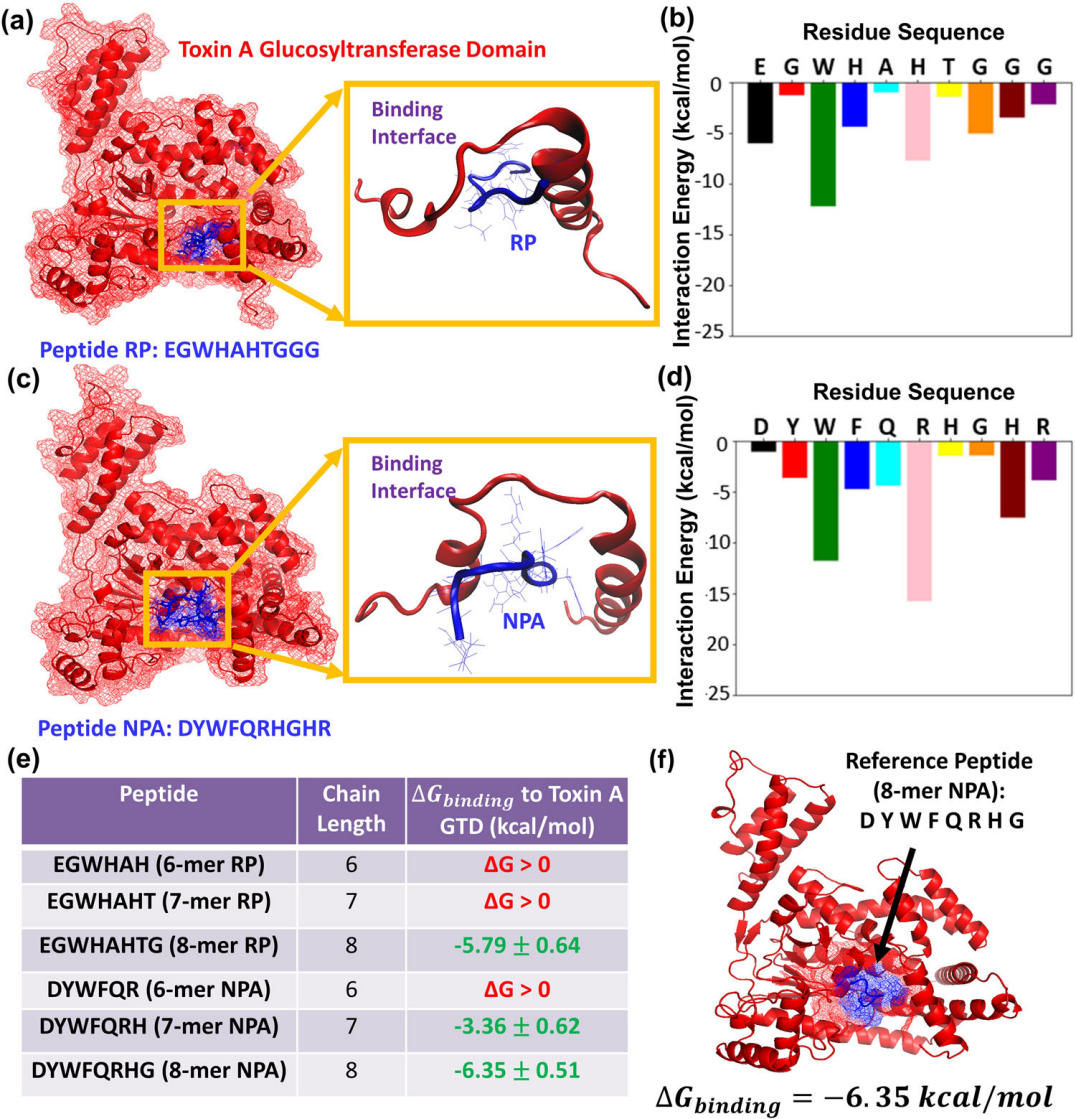
Determining initial peptide sequence to design peptides that will block C. diff. Toxin A in colon cells. **a** Peptide RP (EGWHAHTGGG) at the catalytic site of the Toxin A Glucosyltransferase Domain and (**b**) the residue wise decomposition of the interaction energy plot of peptide RP. **c** Peptide NPA (DYWFQRHGHR) at the catalytic site of the Toxin A Glucosyltransferase Domain and (**d**) the residue wise decomposition of the interaction energy plot of peptide NPA. The TcdA GTD is shown in red, and the peptide is colored in blue. **e** Table showing predictions from molecular dynamics simulation of whether or not the 6-mer, 7-mer, and 8-mer fragments on RP and NPA bind to TcdA GTD (**f**) Starting structure for PepBD: Reference peptide NPA (8-mer) bound to the TcdA GTD at the catalytic site with a ${\triangle G}_{{binding}}$ of −6.35 kcal/mol.

We began by determining which fragment of NPA plays the most important role in binding to the TcdA GTD catalytic domain, as this fragment can then serve as the reference peptide in our design process. NPA was identified in our previous study^39^, which reported computationally designed 10-mer peptide sequences (Supplementary Table 1) that were experimentally tested using a functional cell culture assay for their ability to neutralize TcdA in the small intestinal (jejunum) and large intestinal (colon) cells. The reference peptide used to initialize the computational design in that study was RP: *EGWHAHTGGG* (Fig. 2a), discovered by Feig’s team at Wayne State University^41^ using phage display and verified experimentally by them to inhibit the glucosyltransferase activity of TcdA. Using our PepBD algorithm^26–29^ and molecular dynamics simulations, we identified peptide NPA: *DYWFQRHGHR* (Fig. 2c) that binds to TcdA GTD. The critical residues on RP involved in binding to TcdA GTD are E1, W3, H4 and H6 while the critical residues on NPA involved in binding to TcdA GTD are W3, R6 and H9. The key interacting residues on TcdA GTD are within the reactive loop, viz. residues 509–526. A detailed analysis of the residue-residue interaction between RP:TcdA GTD and NPA:TcdA GTD can be found in our previous work^39^. The amino acid sequences of residues 509–526 are provided in Supplementary Note 1. Peptides RP and NPA showed toxin-neutralizing activity in jejunum cells but showed no effect in the colon cells. Since cells of the small intestine express proteases on the brush boarder of the cell to break down dietary proteins^41^, we speculated that when NPA was applied to jejunum cells, it was getting cleaved into smaller fragments with neutralizing activity. We performed LC-MS/MS on cell culture supernatants from colonic monolayers after peptide NPA and confirmed the presence of shorter peptides derived from full-length NPA. This is not surprising since expression of common proteases in the colon is nearly absent^42^. Thus, based on the assumption that peptides smaller than 10-mers might act as stronger toxin inhibitors, we modified our computational design approach.

Based on the experimental observations described above, we proceeded to test via molecular level simulations if peptides shorter than 10-mers can bind to the TcdA GTD. First, we performed atomistic molecular dynamics simulations of RP and NPA bound to the catalytic domain of TcdA GTD. Plots of the interaction energy (van der Waals + electrostatic + polar solvation energy terms) of each of the ten residues along the peptide sequence with the catalytic site of TcdA GTD were generated. The results indicate that residues on the N-terminus of the peptides have a higher contribution to the interaction energy with the catalytic site of TcdA GTD than the flanking residues on the C-terminus of the peptides (Fig. 2b, d). Second, we simulated 6-, 7-, and 8-mer fragments of peptides RP and NPA bound to the catalytic domain of TcdA GTD. The simulations reveal that the 6-mer and 7-mer fragments of RP, *EGWHAH* and *EGWHAHT* are not stable $(i.e.,\,{{{{{\boldsymbol{\triangle }}}}}}{G}_{{binding}} > 0)$ near the binding site of TcdA GTD, whereas the 8-mer fragment *EGWHAHTG* binds to TcdA GTD with a binding free energy of ${{{{{\boldsymbol{\triangle }}}}}}{G}_{{binding}}=-5.79\frac{{kcal}}{{mol}}$). In contrast, both the 7-mer and 8-mer fragments of NPA, namely 7-mer *DYWFQRH* and 8-mer *DYWFQRHG* (${{{{{\boldsymbol{\triangle }}}}}}{G}_{{binding}}\,{{{{{\rm{for}}}}}}\,8-{{{{{\rm{mer\; NPA}}}}}}=-6.35\frac{{kcal}}{{mol}}$) show good binding affinity for the TcdA GTD (Fig. 2e). Accordingly, we resolved to design 8-mer peptide variants using *DYWFQRHG* as reference ligand for the PepBD algorithm.

### In silico screening of TcdA GTD binding peptides and evaluation of binding free energies

PepBD is a Monte Carlo-based *pep*tide *b*inding *d*esign algorithm that uses an iterative procedure to optimize the binding affinity and selectivity of peptides to a biomolecular target. The algorithm utilizes as input the structure of the complex formed between an initial peptide sequence (reference peptide) and the target biomolecule and selects peptide variants by implementing sequence and conformation change moves on the peptide chain. The desired hydration properties of the designed peptides can be customized based on 6 residue types (hydrophobic, hydrophilic, positive, negative, other, and glycine). The classification of the 20 natural amino acids into the six residue types can be found in Supplementary Table 2. A score function, ${\varGamma }_{{score}}$, which considers (i) the binding energy of the peptide to the receptor and (ii) the conformational stability of the peptide when bound to the receptor, is used to evaluate the acceptance of new peptide candidates. Details of the algorithm are provided in Methods section.

**Fig. 3:**
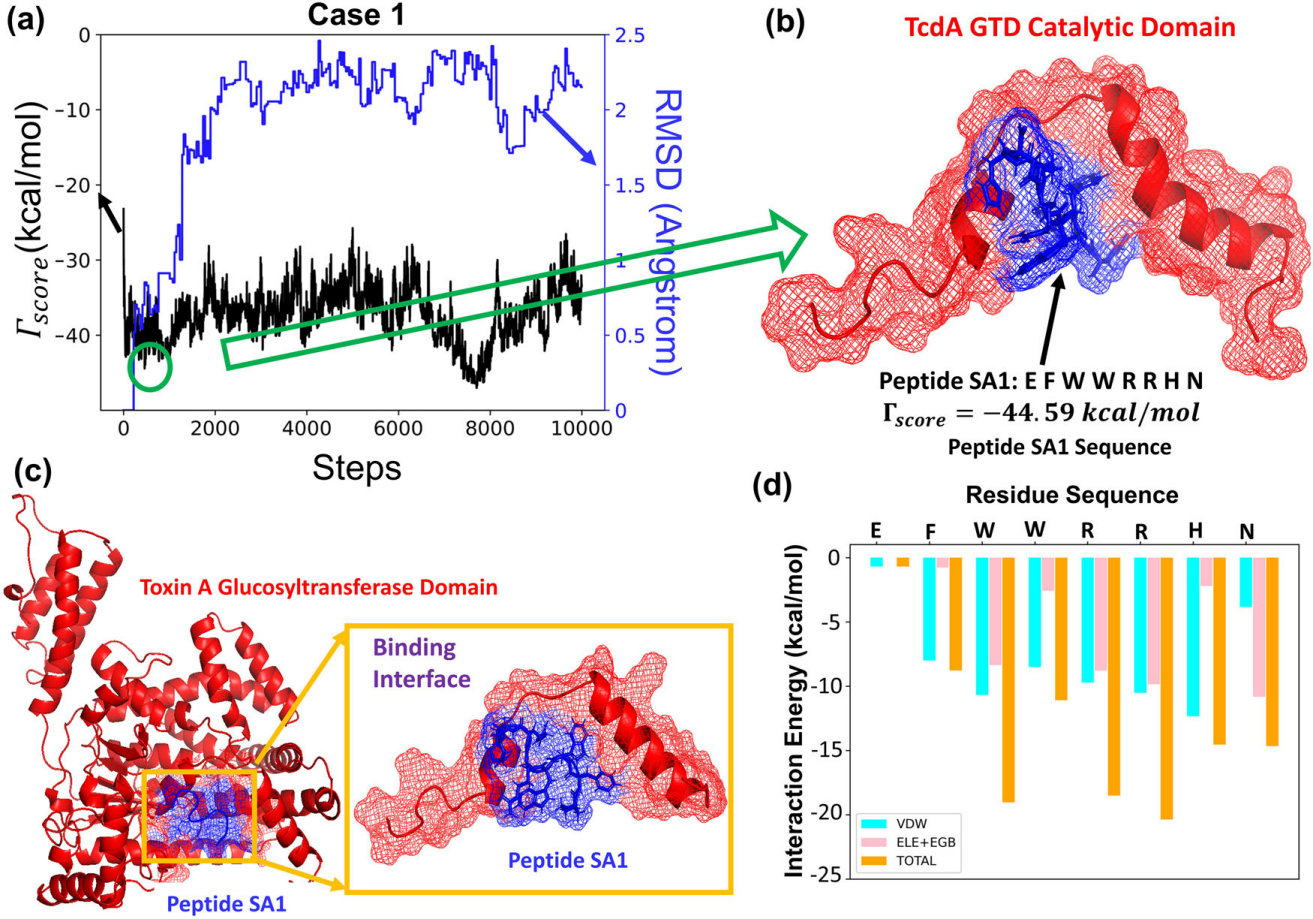
Peptide sequence SA1 was obtained from PepBD and molecular dynamics simulations. **a** The score/RMSD vs the number of sequence and conformation steps for Case 1 with a distinct initial random seed results in (**b**) peptide SA1: EFWWRRHN (**c**) Snapshot of peptide SA1 bound to TcdA GTD obtained from molecular dynamics simulation. The conformation of the peptide at the binding interface is shown. **d** Plot showing the residue-wise decomposition of the interaction energy (van der Waals + Electrostatic + Polar solvation energy contribution) in the SA1:TcdA GTD complex.

We implemented the PepBD algorithm to identify an improved set of peptide inhibitors using the 8-mer NPA:TcdA GTD complex (Fig. 2f) as the input structure. The ${{{{{\boldsymbol{\triangle }}}}}}{G}_{{binding}}$ of 8-mer NPA bound to the TcdA GTD is −6.35 kcal/mol. Our goal is to utilize PepBD to generate new sequences that can bind to the TcdA GTD with higher binding affinity than 8-mer NPA. We investigated three cases with different sets of hydration properties for the peptide chain. For all three cases we ensure diversity in the amino acid composition of the peptide chain by allowing a balance among the various contributions to the binding energy, namely electrostatic, hydrophobic, hydrogen bonding, π-π, etc. which contributes to the peptide’s affinity and selectivity. The three cases are as follows. Case One: *N*_hydrophobic_ = 3, *N*_hydrophilic_ = 2, *N*_positive_ = 2, *N*_negative_ = 1, *N*_other_ = 0 and *N*_glycine_ = 0, Case Two: *N*_hydrophobic_ = 3, *N*_hydrophilic_ = 2, *N*_positive_ = 1, *N*_negative_ = 1, *N*_other_ = 0 and *N*_glycine_ = 1 and Case Three: *N*_hydrophobic_ = 1, *N*_negative_ = 3, *N*_positive_ = 2, *N*_negative_ = 1, *N*_other_ = 0 and *N*_glycine_ = 1. For each case we perform the PepBD search with three different initial random seed numbers to randomize the initial peptide sequence. This enables our designs to proceed along different search pathways and sample peptides from a large pool of peptide sequences and conformations. During the *in-silico* evolution, new sequences and conformers are generated by mutating and exchanging amino acids on the peptide chain, which results in a fluctuation of the score. A lower Γ_score_ means stronger binding affinity of a peptide to the bound target. The root-mean-squared deviation, RMSD, of the new peptide conformers compared to the conformation of the initial peptide chain’s conformation reflects the changes in the backbone scaffold of the peptide as the design process progresses. Fig. 3a shows the Γ_score_ and the RMSD profile vs the number of sequence and conformation change moves performed with a distinct initial random seed for Case 1. Fig. 3b shows the structure of one of the top performing peptides, SA1: *EFWWRRHN*, complexed with the TcdA GTD binding interface from Case 1 (random seed 1). Peptide SA1 has a Γ_score_ = −44.59 which was obtained at the 459^th^ step of the sequence evolution. From Case 2 (random seed 3) the top performing peptide is SA3 *QEWMGRHW* (Supplementary Note 2, Supplementary Fig. 1A, C), and from Case 3 (random seed 3) the top performing peptide is SA6 *EGWQHRHR* (Supplementary Note 2, Supplementary Fig. 1B, D); more information on SA3 and SA6, and Cases 2 and 3 is provided in Supplementary Note 2. A comprehensive list of the top peptide sequences obtained from PepBD, with their corresponding Case, random seed, ${\varGamma }_{{score}}$, ${\triangle G}_{{binding}}$ values and whether or not they were evaluated experimentally is provided in Supplementary Table 3.

Once the *in-silico* evolution terminates, we perform explicit-solvent atomistic molecular dynamics (MD) simulations of the complexes formed between the lowest scoring peptides and TcdA GTD to predict their binding affinity. It is to be noted that ${\varGamma }_{{score}}$ is not an accurate measure of the binding free energy of a peptide bound to TcdA GTD but is rather an important metric for us to use in selecting the peptides that we would like to move forward to perform explicit solvent MD simulations. Three independent simulations are carried out for each peptide:TcdA GTD complex for 100 ns to ensure that the system reaches an equilibrated state. The simulations are performed at 298 K using the AMBER ff14SB forcefield and the AMBER18 package. We calculate the ${\triangle G}_{{binding}}$ of the peptide:receptor complex following the MD simulations using the MMGBSA protocol and variable dielectric constant method . Details of our atomistic MD simulation and ${\triangle G}_{{binding}}$ calculation procedure are provided in *Methods* and Supplementary material^26–29,43^. Table 1 reports the top peptides that we obtain from our computational procedure and their corresponding scores and ${\triangle G}_{{binding}}$ values. We performed a few 500 ns simulations on select peptide:protein complexes and concluded that there were no major conformational changes at long timescales (*see* Supplementary Note 3, Supplementary Fig. 2, Supplementary Tables 4 and 5). SA1 is the most promising peptide with a ${\triangle G}_{{binding}}$ value of −15.94 kcal/mol. (Note: the lower the value of ${\triangle G}_{{binding}}$, the higher the binding affinity). The SA1:TcdA GTD complex obtained by performing a hierarchical clustering analysis on the last 5 ns of a 100 ns MD simulation is shown in Fig. 3c. Additionally, the plot of the residue-wise decomposition of the interaction energy between SA1 and the catalytic site of TcdA GTD is shown in Fig. 3d. The plot reveals that the critical SA1 residues involved in TcdA GTD binding are Trp3, Trp4, Arg5, Arg6, His7 and Asn8. Thus, tryptophan, arginine, histidine, and asparagine on SA1 are the four essential amino acids for TcdA GTD binding. A detailed discussion of key amino acid interactions of SA1 with TcdA GTD is provided in the Supplementary Note 4 and Supplementary Fig. 3. Amino acid sequence signatures in *C. diff* TcdA GTD binding peptides were derived from the residue composition of the top 1% of the lowest scoring peptides identified by PepBD for Cases 1 and 2 (*see* Supplementary Note 5, Supplementary Fig. 4, Supplementary Table 6).

**Table 1:** The initial peptide sequence (8-mer NPA) and the list of peptide sequences identified by PepBD screening with their corresponding ${\Delta \varGamma }_{{score}}$ and ${\Delta G}_{{binding}}$ values with S.E (*n* = 1250 snapshots).

Peptide	Case	Sequence	${\varGamma }_{{score}}\left(\frac{{kcal}}{{mol}}\right)$	${\Delta G}_{{binding}}\left(\frac{{kcal}}{{mol}}\right)$
8-mer NPA		DYWFQRHG		−6.35 ± 0.51
SA1	Case 1	EFWWRRHN	−44.59	−15.94 ± 0.40
SA2	Case 1	QDWMRRHW	−50.20	−13.19 ± 0.39
SA3	Case 2	QEWMGRHW	−43.21	−11.76 ± 0.42
SA4	Case 1	MFWEHRHR	−46.74	−11.01 ± 0.49
SA5	Case 2	EFWMGRHH	−42.83	−6.16 ± 0.45
SA6	Case 3	EGWQHRHR	−44.37	−12.54 ± 0.49
SA7	Case 3	HEWGRRHN	−44.90	−9.56 ± 0.47

### Bead-based pre-screening for TcdA binding

The candidate peptide inhibitors suggested by PepBD were pre-screened in vitro for potent and selective TcdA GTD binding to eliminate weak inhibitors prior to the cell-based assays. Robust inhibition of TcdA glucosyltransferase activity relies on the ability of the peptides to outcompete the TcdA GTD’s substrate, UDP-Glucose. Given TcdA’s relatively low Michaelis constant (K_M_ ~ 4.5 μM) compared to the cellular concentration of its substrate UDP-Glucose (92 μM), it is especially important that the peptides exhibit high binding strength and selectivity to TcdA GTD (see Supplementary material for detailed analysis)^11,44–47^.

**Fig. 4:**
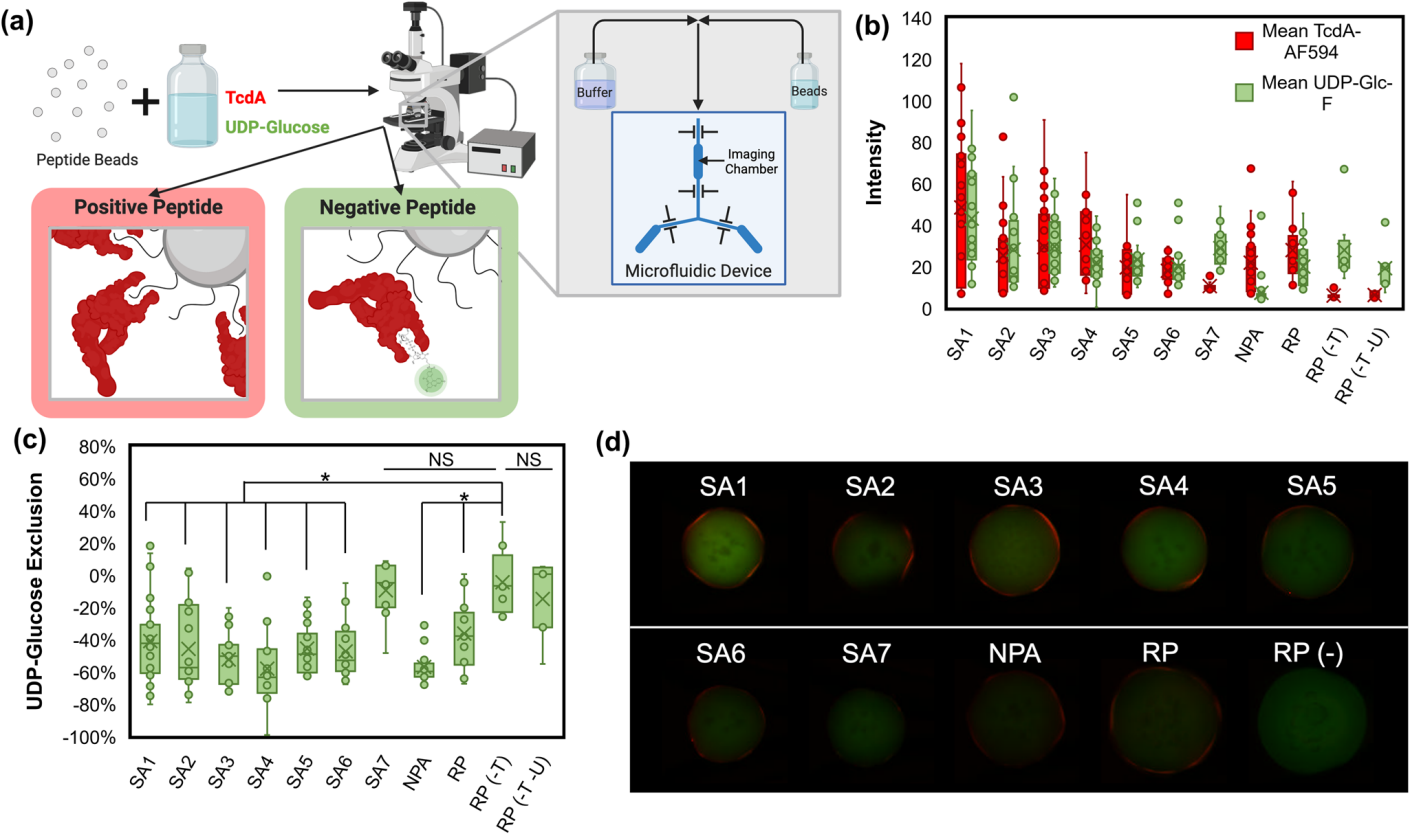
Bead based screening for selective TcdA GTD binding for each peptide sequence using TcdA-AF594 (in red) and UDP-Glucose-Fluorescein (in green). **a** Schematic for bead capture and imaging on custom microfluidic device and microscope. **b** Mean red and green intensity (0–255 pixel value) of 90^th^ percentile red area (TcdA halo) of beads displaying each peptide (number of beads (n) = 23, 20, 25, 19, 23, 21, 15, 36, 14, 8, 7 respectively). RP (-T) and RP (-T -U) are no TcdA-AF594 and no TcdA-AF594/UDP-Glucose-Fluorescein controls, respectively. **c** Percent change in green fluorescence from 90^th^ percentile red halo region to 50^th^ percentile green; more negative values indicate exclusion of UDP-Glucose from peptide:TcdA interface, and thus selective peptide binding to the TcdA GTD (* *p* < 0.005). **d** Representative red and green composite images for each peptide.

Using a microfluidic screening system developed by our team in prior work^36,38^, we implemented a dual-fluorescence assay to evaluate the TcdA GTD inhibitory activity of the peptide candidates SA1-SA7, 8-mer NPA and 8-mer RP (Fig. 4a). Each peptide sequence was immobilized on a translucent ChemMatrix Aminomethyl bead that was then contacted with red fluorescently labeled TcdA (TcdA-AF594) and with a green-fluorescent analog of UDP-Glucose (UDP-Glucose-Fluorescein). Beads displaying peptides that bind to TcdA exhibit a red fluorescence signal; beads exhibit green fluorescence when UDP-Glucose-Fluorescein binds to the catalytic site of the TcdA GTD. Thus, peptides that can selectively bind to the TcdA GTD and displace the UDP-Glucose-Fluorescein from the catalytic site will exhibit red, but not green, fluorescence. Beads are analyzed by a high-throughput assay (350 beads per hr) that correlates the fluorescence intensity of the beads to the binding strength and selectivity of the peptides displayed. Images of each bead are analyzed via a custom algorithm that ensures consistent and objective bead characterization within a screened ensemble.

Beads displaying TcdA- binding peptides accumulate TcdA-AF594 on their surface, leading to a characteristic red halo fluorescence. This is because TcdA is quite large (MW = 308 kDa, 2710 residues) and hence, poorly diffuses into the narrow pores of the beads. The intensity of the halo correlates with the amount of TcdA-AF594 bound^38^ (Fig. 4b). Beads displaying selective TcdA GTD-binding peptides displace UDP-Glucose-Fluorescein from the GTD, and thus peptide:TcdA GTD binding, can be observed as a loss of green fluorescence^44^ from the TcdA binding region (Fig. 4c).

The performance of the peptides (SA1-SA7, 8-mer NPA and 8-mer RP) was inferred based on the intensity of the red halo fluorescence, and on the loss of green fluorescence of the beads, respectively (Fig. 4d). SA1 was the most promising TcdA binding peptide with the highest mean red halo fluorescence. Despite having high green fluorescence, there was a significant (*p* = 0.0011) reduction in the green fluorescence from the center of the beads to the peptide:TcdA binding interface compared to the no-TcdA-AF594 no-UDP-Glucose-Fluorescein control (RP(-T -U)) (Fig. 4c), meaning that SA1 bound selectively to TcdA GTD. SA1, SA2, SA3 and SA4 had higher mean red halo fluorescence, and thus higher TcdA binding, than 8-mer NPA. SA1, SA3 and SA4 had higher mean red halo fluorescence, and thus higher TcdA binding, than 8-mer RP. Notably, all of the peptides that were screened showed binding to TcdA, however the peptides from Cases 1 (SA1, SA2, SA4) and 2 (SA3, SA5) performed significantly better than those from Case 3 (SA6, SA7). All peptides except SA7 showed a significant (*p* < 0.005) decrease in green fluorescence from the center to the halo (TcdA binding) region, indicating relatively universal exclusion of UDP-Glucose from the peptide:TcdA interface (Fig. 4d). Despite differences in background green fluorescence between peptides, the uniform decrease in green fluorescence at the TcdA halo provided little information to differentiate between peptides. The selective binding of most PepBD designed peptides to the TcdA GTD demonstrates the robustness of the peptide design algorithm to reliably identify peptide binders to particular pockets. Peptides SA1-SA4, which had higher red fluorescence than NPA and thus promising TcdA binding, were selected for further evaluation.

### Functional testing of SA1 on human colonic epithelium

**Fig. 5:**
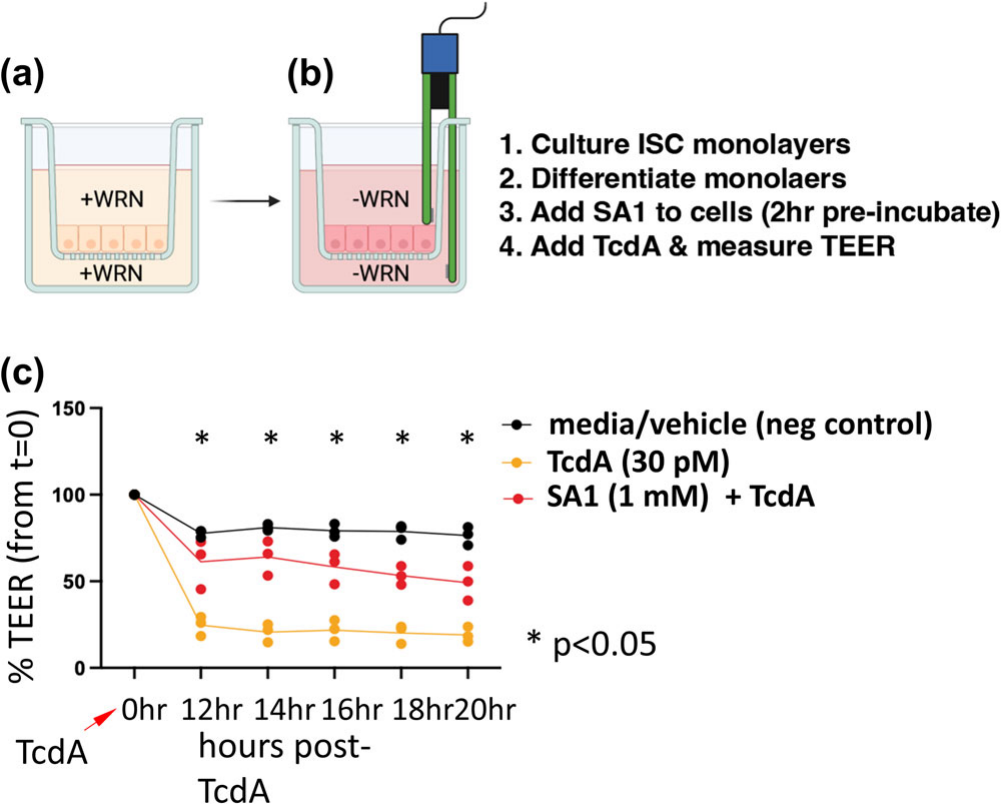
Peptide SA1 has functional neutralizing effects on TcdA in a human colonic epithelial culture model. **a** Summary of experimental design and schematic of human ISC expansion in defined media (EM) of human colonic epithelial monolayers on transwells (**a**). Forced differentiation with defined media (DM) (**b**). **c** TEER measurements on differentiated Colon (Descending) monolayers treated with DM (media with vehicle), TcdA, and TcdA with SA1. Values are expressed as a % of TEER at t = 0 when TcdA was added to cultures. One way ANOVA *p* < 0.005, multiple comparisons tests 12 h-20 h *p* < 0.05.

Peptides SA1-SA4 were screened on a human colon epithelial culture system to preliminarily evaluate neutralizing capabilities. In this assay system, colonic epithelial stem cells are applied to transwell inserts and cultured to confluence on the permeable membrane of the insert (Fig. 5a, b). Once the cell barrier is achieved after about 4 days of ISC (intestinal stem cell) expansion, the media is changed to promote differentiation into the primary absorptive lineage of the colon and the cell type most exposed to C. difficile toxins^48–50^. Tight junction proteins are upregulated thereby increasing the barrier function of the cellular monolayer and decreasing the ion flux, which is measured as Trans-Epithelial Electrical Resistance (TEER)^51^. Toxicity is measured over time as a drop in TEER indicating TcdA-dependent changes in cytoskeleton, which cause leaky tight junctions. For the quick screen, peptides were pre-incubated with differentiated colon epithelial monolayers for 2 hours, then TcdA was added at a concentration of 30 pM, which is the concentration observed in stool of *C. diff* patients^51^. SA1 demonstrated some neutralizing capabilities in the primary screen as observed by preservation of TEER compared to conditions with TcdA without SA1. Because SA1 showed promising neutralizing activity, it was more extensively tested for efficacy using the same TcdA toxicity assay. As expected, colon monolayers responded with a near complete loss of TEER at 12 hours post-TcdA exposure, however, pre-exposure of monolayers with SA1 produced ~79% protection from TcdA toxicity (Fig. 5c). These data demonstrate the ability of SA1 to protect barrier function in the presence of clinically relevant concentrations of TcdA.

### Measuring SA1 kinetic parameters by surface plasmon resonance

**Fig. 6:**
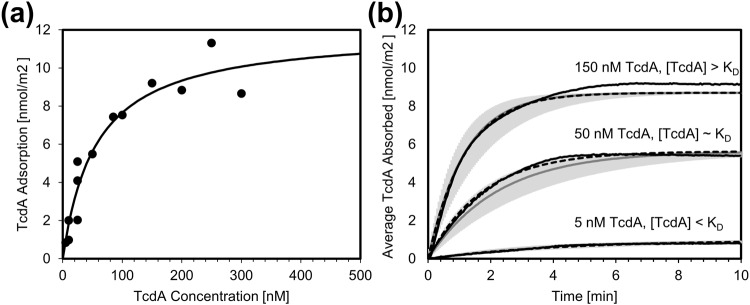
Surface plasmon resonance adsorption isotherms and dynamic responses for SA1:TcdA binding. **a** Maximum TcdA adsorption and TcdA concentration fit to a Langmuir isotherm (Eq. 1) yielding K_D_ and Q_max_. **b** Experimental dynamic response (black solid lines), individual theoretical fit (black dashed lines), and average theoretical fit (solid grey lines, 95% confidence interval in light grey) for TcdA concentrations above, below, and approximately at K_D_.

Surface plasmon resonance was used to measure key kinetic parameters for SA1:TcdA binding. SA1 was covalently bound to a mixed bio-resistant thiol self-assembled monolayer (SAM) on the gold sensor surface. Characterization of the surface thickness and composition can be found in Supplementary Note 6, Supplementary Figs. 5–7. Various concentrations of the analyte, TcdA, were injected over the surface and the net angular response in degrees was recorded. The amount of analyte, TcdA, bound to the surface was calculated using a device-specific conversion factor previously described^52^. The net equilibrium response was fit to a Langmuir isotherm (Eq. 1) as shown in Fig. 6a, resulting in an equilibrium dissociation constant, K_D_, of 56.1 ± 29.8 nM and a maximum binding capacity, Q_max_, of 12.0 ± 2.2 nmol/m^2^.

1 $$Q=\frac{{Q}_{\max }\left[{TcdA}\right]}{{K}_{D}+\left[{TcdA}\right]}$$

SA1:TcdA binding is a moderate-to-high affinity interaction based on its mid-nanomolar K_D_. A fuller picture of the binding emerges by considering the kinetics in addition to the equilibrium state. Rapid recognition, which is characterized by a high adsorption rate constant (k_a_), is important in the context of competitive inhibitors. Additionally, complex stability, as reflected by a low desorption rate constant (k_d_), is desired. To succeed as a potent competitive TcdA inhibitor, SA1 needs to outcompete UDP-Glucose for the active site and slowly dissociate from it. Dynamic response measurements over TcdA injections allowed for the calculation of k_a_ and K_D_ using Eq. 2, which are summarized in Table 2, and shown in Fig. 6b.

2 $${Q}_{t}=\frac{{Q}_{\max }\left[{TcdA}\right]}{{K}_{D}+\,\left[{TcdA}\right]}\left(1-\frac{1}{{e}^{\left(\left[{TcdA}\right]+{K}_{D}\right){k}_{a}t}}\right)$$

**Table 2:** Association and dissociation constants for various TcdA concentration ranges.

Concentration Range	${Average}\;{k}_{a}\;{at\; flow\; rate}\;30\frac{\mu l}{\min }\left[\frac{n}{{M\; s}}\right]$	${Average}\;{k}_{d}\;{at\; flow\; rate}\;30\frac{\mu l}{\min }\left[\frac{1}{s}\right]$
[TcdA] < K_D_	8.1 × 10^−5^	4.6 × 10^−3^
[TcdA] ~ K_D_	7.1 × 10^−5^	4.0 × 10^−3^
[TcdA] > K_D_	5.4 × 10^−5^	3.0 × 10^−3^
All [TcdA]	7.0 ± 2.5 × 10^−5^	4.0 ± 1.4 × 10^−3^

The average k_a_ was found to be 7.0 ± 2.5 × 10^−5^ nmol^−1^ s^−1^, which is high for a peptide inhibitor, given its relative backbone flexibility. Review of the literature indicates that peptide inhibitor adsorption rate constants typically fall between 10^−7^ and 10^−6^ nmol^−1^ s^−1^ ^53,54^. The average k_d_ was found to be 4.0 ± 1.4 × 10^−3^ s^−1^, which is consistent with that of other peptide ligands^53–57^. Compared to antibody:antigen binding interactions, which are known to be high affinity (typical K_D_ are between 10^−8^ and 10^−11^ M), SA1:TcdA (K_D_ ~ 10^−8^ M) exhibits remarkably high binding affinity for non-antibody:antigen biorecognition^58^.

## Discussion

The goal of this work was to identify lead peptide candidates that bind to the catalytic site of TcdA glucosyltransferase domain and hence can inhibit the glucosyltransferase activity of toxin TcdA. In our previous work, we reported 10-mer peptides that were able to neutralize TcdA in differentiated small intestinal absorptive cells (SI) but showed no effect on differentiated colon absorptive cells. A likely explanation for this observation is that proteases present on the brush border of SI cells cleaved the 10-mer peptides into shorter, more-active forms that neutralize the toxins in the SI cells. To probe this possibility, we performed mass spectrophotometry on the media containing the 10-mer reference peptide (EGWHAHTGGG^40^) that had been incubated with SI cells. After 20 hours of exposure to the SI cells the reference peptide showed 4 predominant and smaller peptides and the full-length 10-mer reference peptide was the least abundant species suggesting the 10-mer was being cleaved by SI proteases (Supplementary Fig. 8). We would not expect this effect in colon cells as they do not generally express proteases^42^. We performed atomistic molecular dynamics simulations to test if shorter peptides might be more effective inhibitors of *C. diff*. TcdA than the 10-mers. The simulations predicted that 8-mer peptides are at an optimum peptide length. We applied our PepBD algorithm and combined it with molecular dynamics simulations and binding free energy calculations to find seven 8-mer peptides (SA1-SA7). These peptides were rapidly screened using an in-house bead-based microfluidic screening technique to check for selective TcdA- GTD binding, and the weak peptide inhibitors (SA5-SA7) were eliminated. The efficacies of peptides SA1-SA4 were tested using a trans-epithelial electrical resistance (TEER) assay on monolayers of the human gut epithelial stem cells from the large intestine (descending colon). Peptide SA1 blocked TcdA toxicity in colon epithelial cells. The binding affinity of this peptide to TcdA was characterized using surface plasmon resonance (SPR) and the dissociation constant, K_D_, was found to be 56.1 ± 29.8 nM.

One of our future goals is to develop peptide inhibitors that bind at the catalytic site of TcdB GTD, as TcdB is an even more challenging and clinically relevant target than TcdA. We computationally evaluated the binding affinity of SA1 for the TcdB GTD (method summarized in the Supplementary Note 7). Our computational predictions suggest that peptide SA1 exhibits low binding affinity (${{{{{\boldsymbol{\triangle }}}}}}{G}_{{binding}}=-1.56{kcal}/{mol}$) for the TcdB GTD catalytic site. Hence, SA1 is selective towards Tcd A.

Anti-toxin drugs (such as SA1 developed here) are therapeutically relevant because they eliminate the causative agent of disease (e.g., the toxin). Anti-toxin drugs are also appealing because they are highly specific (limiting off-target effects on the host or microbiota) and impose low, if any, fitness cost on the toxin-producing pathogen (thereby reducing the pressure for resistance to develop). In this way, anti-toxin drugs are complementary to (and potentially synergistic with) standard-of-care antibiotics. By binding to the toxin’s catalytic site, SA1 competitively inhibits the key disease-causing biochemical reaction employed by *C. diff*. Further, because the toxin active site is the most highly conserved region of the toxin, we hope that SA1 will be active on a variety of *C. diff* strains and maintain robust activity as new strains arise. SA1 represents an improvement in anti-TcdA GTD peptide therapeutics that is similarly potent, but mechanistically distinct from recently published small molecule glucosyltransferase inhibitors^59,60^. Looking forward, the ease with which peptides can be manufactured at scale promises to improve the equitability of access to anti-toxin therapies, and also allows them to be manufactured at the site of disease via engineered gut microbes. Taken together, this work illustrates a structure-guided, rational approach to designing anti-toxin peptides that is readily generalizable to other toxins secreted by *C. diff* and other antibiotic-resistant pathogens.

## Methods

### Materials

N, N-dimethylformamide (DMF), dichloromethane (DCM), Alexa Fluor 594 NHS Ester, N-hydroxysuccinimide (NHS), 1-ethyl-3-(3-dimethylaminopropyl) carbodiimide hydrochloride (EDC), sodium chloride, HPLC-grade acetonitrile, HPLC-grade formic acid, LCMS-grade acetonitrile, LCMS-grade formic acid, Pierce™ Dye Removal Columns, glacial acetic acid, and hydrochloric acid were purchased from ThermoFisher Scientific (Waltham, MA). Triisopropylsilane-silane (TIS), sodium hydroxide (NaOH), phosphate-buffered saline (PBS) pH 7.4, 1,2-ethanedithiol (EDT), Tween 20, thioanisole, phenol, 3 kDa MWCO Amicon Ultra centrifugal filters, and Glucose-UDP-Fluorescein conjugate were purchased from Millipore-Sigma (Burlington, MA). 30% Hydrogen peroxide, Kaiser test kits, and Magnesium chloride were purchased from Sigma Aldrich (St. Louis, MO). 96% sulfuric acid was purchased from Macron Fine Chemicals (Randor, PA). Petroleum ether, and ethyl ether were purchased from EMD Millipore Corporation (Darmstadt, Germany). Tris HCl was purchased from IBI Scientific (Peosta, IA). Piperidine, Diisopropylethylamine (DIPEA), trifluoroacetic acid (TFA), 1-methyl-2-pyrrolidinone (NMP), 2-(7-aza-1Hbenzotriazol-1- yl) −1,1,3,3-tetramethyluronium hexafluorophosphate (HATU), Fmoc-L-Arg(Pbf)-Wang resin, Fmoc-L-Trp(Boc)-Wang resin, Fmoc-L-Thr(tBu)-Wang resin, Fmoc-L-Pro-Wang resin, Fmoc-L-Asn(Trt)-Wang resin, Rink-Amide resin, and all Fmoc protected amino acids were purchased from ChemImpex, Inc. (Wood Dale, IL). ChemMatrix Aminomethyl resin (0.7 mmol/g functional density, 100–200 mesh) was purchased from PCAS Biomatrix, Inc. (Saint-Jean-sur-Richelieu, Quebec, Canada). Toxin A from Clostridioides difficile was purchased from List Biological Labs, Inc. (Campbell, CA). Bioresistant alkanetiols hydroxyl terminated (HSC_11_(EG)_3_OH, 2-{2-[2-(1-mercaptoundec-11-yloxy)-ethoxy]-ethoxy}-ethanol) and carboxyl terminated (HSC_11_(EG)_6_OCH_2_COOH, (2-(2-(2-(2-(2-(2-(11-mercaptoundecyloxy)-ethoxy)-ethoxy)-ethoxy)-ethoxy)-ethoxy-acetic acid)) were obtained from Prochimia Surfaces (Poland). Gold sensor slides were obtained from BioNavis Ltd. (Tampere, Finland). Ethanol (200 proof) was obtained from Decon Labs, Inc (King of Prussia, PA). Milli-Q water (MQ water, resistivity > 18 MΩ cm) was obtained by using a Millipore water purification system (Billerica, MA). Nitrogen gas and liquid nitrogen were obtained from Airgas National Welders (Raleigh, NC).

### Computational peptide design

The PepBD algorithm uses an iterative procedure that optimizes peptide sequences to bind with higher affinity and specificity to a biomolecular target than a known reference ligand. The design process is summarized below.

- *Generate input peptide: TcdA GTD structure:* The input structure for the PepBD algorithm was an 8-mer fragment of an *in-silico* peptide, NPA, identified earlier and experimentally-verified to neutralize TcdA in jejunum cells complexed with the TcdA GTD.
- *Compute initial score of random peptide: TcdA structure:* A random peptide sequence is generated and draped on the backbone scaffold of the initial peptide (NPA 8-mer) bound to the TcdA GTD and its ${\varGamma }_{{score}}$ is calculated.
- *Iteration of peptide sequence-change and conformation-change moves:* The design algorithm performs 10,000 evolution steps and generates variants of the original peptide that bind to the target protein by two kinds of moves: sequence change (mutation) and conformation change.
- *Evaluate score* ${\varGamma }_{{score}}$ *of new peptide sequence/conformer*: The score of the newly generated peptide sequence or conformer in complex with the TcdA GTD is evaluated. The score function, *Γ*_*score*_, that we use to evaluate newly generated peptide candidates is given by:
  3 $${\varGamma }_{{score}}=\Delta {E}_{{binding}}+\lambda \left({E}_{{peptide}-{VDW}}^{{bound}}+{E}_{{peptide}-{ELE}}^{{bound}}+{E}_{{peptide}-{EGB}}^{{bound}}\right)$$
  The first term of Eq. (3), $\Delta {E}_{{binding}}$, accounts for the difference in the energy of the complex and the energies of the peptide and target biomolecule prior to binding. The second term is the peptide stability term and accounts for the energy of the free peptide in the bound-state configuration. λ is a weighting factor for the peptide stability term with a value of 0.01. Lower scores mean better binders. The force field parameters are taken from the Amber 14SB force field.
- *Monte Carlo Metropolis Algorithm:* The Monte Carlo Metropolis algorithm is used to accept or reject new trial peptides.

More details regarding the PepBD algorithm and ${\varGamma }_{{score}}$ can be found in our previous work^26–29^.

### Atomistic molecular dynamics simulation

Explicit-solvent atomistic MD simulations are carried out in the canonical (NVT) ensemble using the AMBER 18 package to investigate the dynamics of the binding process between the peptide sequences and the TcdA GTD. The starting configurations of the peptide:TcdA GTD complexes in each MD simulation are the output from the searches in the PepBD algorithm. We carry out three independent simulations for each peptide:TcdA GTD complex for 100 ns to ensure that the system reaches an equilibrated state. Each peptide-receptor complex is solvated in a periodically-truncated octahedral box containing a 12 Å buffer of TIP3P water ($\sim$ 36,000 water molecules) surrounding the complex in each direction. Counterions such as Na+ or Cl- were added to neutralize the peptide:protein complex prior to running the MD simulations. No additional salt ions were added. The implicit-solvent molecular mechanics/generalized Born surface area (MM/GBSA) approach with the variable internal dielectric constant model is used to post-analyze the last 5 ns simulation trajectories of the peptide:TcdA GTD complexes to calculate the binding free energies. Details of the computational procedures and post-analysis of the atomistic MD simulations can be found in our previous work^26–31^. MD simulation parameters are described in Supplementary Table 7.

### Bead-based screening assay

#### Solid phase peptide synthesis

SA1-SA7, NPA (8-mer), and RP (8-mer) were synthesized on ChemMatrix Aminomethyl resin following a GSG linker on a Biotage Syro I peptide synthesizer (Biotage, Uppsala, Sweden) following the Fmoc/tBu protecting strategy. The GSG linker aids in displaying the peptide on the surface of the resin. The resin was swelled in DMF for 30 min. Amino acids were coupled by incubating the resin with 3 equivalents (relative to functional density of resin) protected amino acid, 3 eq. HATU, and 6 eq. DIPEA in dry DMF for 15 minutes at 45  °C. The coupling of each amino acid coupling was monitored by Kaiser test. Fmoc removal of the resin and after each amino acid conjugation was performed using 5 ml 20% v/v piperidine in DMF at room temperature for 3 min and then again for 10 min. Protected resin was stored dried under nitrogen at 4 °C. The completed peptide was deprotected immediately before use through acidolysis by incubating the resin in the deprotection cocktail, Reagent K, 82.5% v/v TFA, 5% v/v phenol, 5% v/v water, 5% v/v thioanisole, 2.5% v/v EDT for 3 hr at room temperature and under mixing. The deprotected resin was rinsed with DCM, DMF, then DCM and dried under nitrogen.

#### Screening

Peptide sequences were screened for binding to TcdA on an in-house microfluidic bead imaging system originally designed for sorting solid phase peptide libraries^36,38^. Exclusion of a fluorescent UDP-Glucose co-factor analog (UDP-Glucose-Fluorescein) from the UDP-Glucose binding pocket of TcdA was used as a proxy for visualizing peptide binding in the desired position.

Tris-buffer was removed from the TcdA solution with 3 kDa MWCO Amicon Ultra centrifugal filters, through 5 rounds of 10-fold concentration and dilution following the manufacturer’s recommended protocol. TcdA was labeled with Alexa Fluor 594 through NHS chemistry. 1 μl 10 mg/ml NHS-Alexa Fluor 594 was added to 100 μl 1 mg/ml TcdA. After 1 h incubation, unbound dye was removed with Pierce Dye Removal Columns per the manufacturer’s instructions. UDP-Glucose-Fluorescein was dissolved at 1 mg/ml in PBS, 10 mM Magnesium Chloride (Binding Buffer, BB).

Resin was incubated with 0.2 μM fluorescently labeled TcdA (TcdA-AF594) overnight. Immediately prior to screening, UDP-Glucose-Fluorescein was spiked into the resin solution at a final concentration of 0.5 μM for 15 min. The resin was gently washed 4 times with BB + 0.2% Tween 20 (Screening Buffer, SB). Resin beads were imaged in the red and green channels on the microfluidic system previously developed^36,38^. Beads were visualized on an Olympus IX81 Motorized Trinocular Inverted Fluorescence Phase Contrast Microscope fitted with FITC and RFA8 Chroma filter cubes and were imaged with a Hamamatsu C13440 camera. Each resin solution was diluted in excess SB prior to loading on the microfluidic device. Resin beads were flown through the imaging chamber one at a time and imaged in the red (TcdA-AF594) and green (UDP-Glucose-Fluorescein) channels. Approximately 30 individual beads from each peptide sequence were imaged.

Image processing and analysis was performed on the red and green channel images for each bead. Because the red channel displayed prominent halo fluorescence, the 90th percentile of pixels in the bead were used for the analysis. This reduced bias from variance in the center of the bead. Additionally, the concentration of TcdA used for incubation was previously tuned to allow for a range of intensities in the halo, which allowed for differentiation between moderate and strong binders. The mean green fluorescence of the 90^th^ percentile red area and the mean 50th percentile green fluorescence were calculated to determine UDP-Glucose exclusion from TcdA. The average and standard deviation of the 90th percentile of the red channel and the green channel were computed.

### Free peptide synthesis and purification for functional testing of SA1 on human gut epithelium

SA1-SA4 were synthesized on Wang resin pre-loaded with the first amino acid on an Initiator+ Alstra (Biotage, Uppsala, Sweden) following the Fmoc/tBu protecting strategy. The pre-loaded Wang resin was end capped with 1 ml 5 M acetic anhydride in 2.5 ml 2 M DIPEA for 30 minutes at room temperature. Amino acids were coupled by incubating the resin with 5 equivalents (relative to functional density of resin) protected amino acid, 5 eq. HATU, and 10 eq. DIPEA in dry DMF for 5 minutes at 75  °C. The coupling of each amino acid coupling was monitored by Kaiser test. Fmoc removal after each amino acid conjugation was performed using 5 ml 20% v/v piperidine in DMF at room temperature for 3 min and then again for 10 min. The completed peptide was cleaved and side chain protecting groups were removed through acidolysis by incubating the resin in the deprotection cocktail, Reagent K, 82.5% v/v TFA, 5% v/v phenol, 5% v/v water, 5% v/v thioanisole, 2.5% v/v EDT for 3 h at room temperature and under mixing. The deprotected peptide dissolved in the deprotection cocktail was precipitated in ice cold 50% v/v ethyl ether and 50% v/v petroleum ether (ether solution). The solution was cooled at −80  °C for 30 min, pelleted, and washed three times with ice cold ether solution. The crude peptide pellet was dried under nitrogen, redissolved in 50% v/v acetonitrile and 50% v/v water, and dried on the Biotage V-10 Touch (Biotage, Uppsala, Sweden) prior to purification and analysis.

SA1-SA4 were purified via flash chromatography on an Isolera Prime (Biotage, Uppsala, Sweden) with a Biotage Sfär Bio C18 column. Crude peptide was dissolved in 10% v/v acetonitrile, 90% v/v water, and 0.1% v/v formic acid and applied to a column samplet. Reverse phase chromatography was performed with a gradient from 5% to 70% acetonitrile in water. 0.1% formic acid was used as a modifier. Fractions were collected above a threshold 220 nm and 280 nm absorbance. Fractions were analyzed by LC-MS to identify and quantify the desired peptide. Purified peptide fractions were lyophilized. For final polishing, the peptides were dissolved in 50% v/v water and 50% v/v acetonitrile, filter sterilized, aliquoted, and lyophilized.

### Cell-based assay

#### Culture of primary colonic stem cells

Donor Selection: Human transplant-grade donor intestines were obtained from HonorBridge (Durham, NC) and exempted from human subject’s research by the UNC Office of Human Research Ethics. Donor acceptance criteria were as follows: age 65 years or younger, brain-dead only, negative for human immunodeficiency virus, hepatitis, syphilis, tuberculosis, or COVID-19, as well as no prior history of severe abdominal injury, bowel surgery, cancer, or chemotherapy. Colonic tissue from a 34-year-old Hispanic male was used for all studies. Colonic ISCs from this donor were isolated from primary tissues. Surgical specimens of human colon were obtained from donors at HonorBridge (Durham, NC). Crypts from the colon were removed from the specimen by incubation in a chelating buffer for 75 min at 20 °C followed by vigorous shaking in a 50 mL conical tube. The chelating buffer was composed of EDTA (2 mM), dithiothreitol (DTT, 0.5 mM, freshly added), Na_2_HPO_4_ (5.6 mM), KH_2_PO_4_ (8.0 mM), NaCl (96.2 mM), KCl (1.6 mM), sucrose (43.4 mM), D-sorbitol (54.9 mM), pH 7.4. Liberated crypts were expanded as a monolayer on a neutralized collagen hydrogels. Crypts were placed on the top of 1 mg/mL collagen hydrogels (1 ml into each well of 6-well plate) at a density of 10,000 crypts/well, overlaid with 3 mL of Expansion Media (EM)^61^ containing 10 mmol/L Y-27632 (S1049; SelleckChem, Houston, TX), and incubated at 37 °C in 5% CO_2_. EM was used to expand the epithelial cell numbers as monolayers; media was changed the day after seeding and every 48 hours afterwards. When the cell coverage was greater than 80% (typically 4 to 6 days), the epithelium was dissociated to fragments^62^ to seed onto either 6-well tissue-culture plates coated with collagen hydrogels for continued expansion, or onto 12-well Transwell inserts (3460; Corning, Corning, NY) coated with 1% Matrigel for experiments^61^. To generate differentiated enterocytes for the toxicity assays, 2 wells of a 6-well colonic ISC expansion plate, where cells were ~90% confluent, were dissociated^61^ as described above and plated on 12-well transwell inserts coated with 1% Matrigel. Colonic ISCs were expanded in colon expansion media (EM, see Ref. ^61^ for all media formulations) until confluent (~4-days). After colonic ISCs were confluent, the media was changed to differentiation media (DM) and transepithelial electrical resistance (TEER) was measured every 24-hours using the EVOM2 (World Precision Instruments, FL). At 3–4 days of differentiation when TEER was >1000 ohms/cm^2^, the toxicity assays were initiated. All cells were incubated at 37 °C in a humidified environment containing 5% CO_2._

#### TcdA and SA1 exposure to differentiated human colonic epithelial cells

Peptides were diluted at a concentration of 1.0 mM in 500 µl of DM and added to the apical reservoir. The peptides were allowed to preincubate with the colonic monolayers for 2 hours prior to the addition of TcdA (SML1154; Sigma-Aldrich, St. Louis, MO). TcdA at 30 pMol final concentration was added directly to the apical reservoir media containing the peptide and incubated at 37 °C in a humidified environment containing 5% CO_2_. TEER was measured before and after the peptides were applied. TEER was then measured at the timepoints indicated.

### Surface plasmon resonance (SPR)

#### Synthesis and purification of modified SA1 for SPR experiments

Modified peptide SA1-K-amide was synthesized to facilitate grafting to gold sensors through the C-terminal lysine residue. Amide functionalization of the C-terminus prevented electrostatic repulsion between the C-terminus of the peptide and carboxylic acid groups on the SAM. SA1-K-amide was synthesized on Rink Amide resin on an Initiator+ Alstra (Biotage, Uppsala, Sweden) following the Fmoc/tBu protecting strategy as described above. A modified deprotection cocktail, 91.5% w/w TFA, 2.5% w/w water, 2.5% w/w TIS, 2.5% w/w DTT, and 1% w/w indole, was used. SA1-K-amide was collected, purified, and analyzed through the protocol described above.

#### Cleaning gold sensors

Gold sensor slides (12 × 20 × 0.5 mm^3^), 50 nm gold adhered on glass sensors with 2 nm chromium, were cleaned prior to use by soaking in Piranha solution (98% H_2_SO_4_ and 30% H_2_O_2_ at 3:1 v/v) for 15–20 min, followed by profuse rinsing in MQ water, rinsing in 200 proof ethanol, and drying under a stream of nitrogen. The gold sensor slides were briefly immersed in 200 proof ethanol prior to modification. (*Warning: Piranha solution reacts violently with organic materials and should be handled with extreme caution.)*

#### Self-assembled monolayer (SAM) on gold surfaces followed by peptide grafting

A mixed thiol SAM was produced by dissolving HSC_11_(EG)_3_OH and HSC_11_(EG)_6_OCH_2_COOH at a 3:1 molar ratio, 1 mM total concentration, in 200 proof ethanol. The gold sensors were immersed in the thiol solution under nitrogen and protected from light for 24 h. The surfaces were vigorously rinsed with 200 proof ethanol to disrupt multilayers, and then dried under nitrogen for subsequent characterization, peptide grafting, or SPR experiments. Modified peptide SA1-K-amide was grafted to carboxylic acid groups on the SAM through NHS/EDC chemistry. 200 mM EDC and 50 mM NHS dissolved in MQ water was applied to the surfaces of the gold sensor for 1 h. The activated gold sensors were rinsed in MQ water and dried under nitrogen. 1 mg/ml peptide SA1-K-amide was dissolved in MQ water and conjugated to the activated gold sensors for 1 h. The peptide grafted gold sensors were rinsed in MQ water and dried under nitrogen. Remaining activated carboxylic acid sites were blocked by incubating 3 M ethanolamine in MQ water on the surface of the gold sensors for 30 min. The gold sensors were rinsed in MQ water and dried under nitrogen for subsequent characterization and SPR experiments.

#### Surface density of SAMs and grafted peptide

The surface thickness of the mixed thiol SAM and SAM with grafted peptide SA1 was measured using an M-2000 DI Spectroscopic Ellipsometer (J.A. Woollam, Lincoln, NE) at three angles of incidence Φ = 55°, 65°, 75°. From the bottom up, the samples were modeled as 3 mm Cauchy substrate (glass), 2 nm chromium, and 50 nm gold per the manufacturer’s specifications. The initial value of the Cauchy substrate (polymer layer) on the gold surface was estimated to be 25 nm and the surface thickness was measured after the model was generated. Absorbance interference was mitigated by condensing the wavelength range from 600–1000 nm.

#### Time-of-flight secondary ion mass spectrometry (ToF-SIMS) analysis of SPR Sensors

Time-of-flight secondary ion mass spectrometry (ToF-SIMS) characterization of gold sensor slides with self-assembled monolayers (SAMs) and SA1 covalently grafted to SAM. Experiments were performed on an TOF SIMS V instrument (ION TOF, Inc., Chestnut Ridge, NY). Positive and negative spectra were recorded for the mixed-thiol SAM and SAM-SA1. The positive secondary ion mass spectra were calibrated using H^+^, C^+^, C_2_H_3_^+^, C_3_H_5_^+^, and C_4_H_7_^+^. The negative secondary ion mass spectra were calibrated using C^-^, O^-^, OH^-^, and C_n_^-^, respectively.

#### Surface plasmon resonance (SPR) experiments

A KSV SPR 200 instrument (BioNavis Instruments, Helsinki, Finland) was used to detect changes in the refractive index at the sensor interface. Sensors were equilibrated with 30 μl/min 50 mM Tris, 50 mM NaCl, 10 mM MgCl_2_ (Running Buffer, RB) for more than 5 minutes. After a stable baseline was established, 250 μl TcdA in RB at various concentrations was injected at 30 μl/min followed by RB. New sensors were used for each measurement due to the difficulty disrupting the peptide:TcdA binding interaction.

#### Equilibrium and kinetic parameters

The equilibrium dissociation constant, K_D_, was found by measuring the equilibrium net change in SPR signal (degrees) after each injection of TcdA at concentrations from approximately 0.1K_D_ to 10K_D_. The net change in degrees was converted to mass of protein adsorbed per unit area through a previously measured conversion factor, 1 degree response = 7.31 mg/m^2^. The data was fit to a Langmuir isotherm using the adsorbed mass per unit area, Q (nmol/m^2^), and the solution TcdA concentration, [TcdA] (nM),

4 $$Q=\frac{{Q}_{\max }\left[{TcdA}\right]}{{K}_{D}+\left[{TcdA}\right]}$$

where K_D_ is the equilibrium dissociation constant (nM) and Q_max_ is the maximum binding capacity (nmol/m^2^). A Langmuir model is appropriate when there is a 1:1 interaction between the ligand and analyte, there are no mass transfer limitations, and binding events are independent. Assuming reversible binding,

5 $$TcdA+SA1 \mathop{\rightleftharpoons }\limits_{{k}_{d}}^{{k}_{a}} TcdA\cdot SA1$$

where k_a_ is the second order association (adsorption) constant and k_d_ is the first order dissociation (desorption) constant. Assuming no mass transfer limitations, the concentration of TcdA in the bulk and at the surface is equal. The SPR response and amount adsorbed, Q, are proportional to the concentration of TcdA bound to the peptide, [TcdA•SA1]. The maximum SPR response and maximum binding capacity, Q_max_, are proportional to the maximum bound ligand [TcdA]_tot_. Substituting these into the rate equation yields,

6 $$\frac{{dQ}}{{dt}}={k}_{a}\left[{TcdA}\right]\left({Q}_{\max }-{Q}_{t}\right)-{k}_{d}{Q}_{t}$$

Integrating Eq. 6 and substituting for k_d_,

7 $${Q}_{t}=\frac{{Q}_{\max }\left[{TcdA}\right]}{{K}_{D}+\,\left[{TcdA}\right]}\left(1-\frac{1}{{e}^{\left(\left[{TcdA}\right]+{K}_{D}\right){k}_{a}t}}\right)$$

The first term in Eq. 7 determines the equilibrium level, and the second term determines the time to reach equilibrium. The dissociation constant can be determined from K_D_ and k_a_.

### Statistics and reproducibility

Details of the experiments are provided in the main text and in “Methods”. For the bead-based assay, statistical analyses were performed using a two-tailed T-test in conjunction with the RP (-T) control. Significance was determined at *p* < 0.05. The number of replicates for each sample was as follows: SA1 (23), SA2 (20), SA3 (25), SA4 (19), SA5 (23), SA6 (21), SA7 (15), NPA (36), RP (14), RP (-T) (8), and RP (-T -U) (7). Approximately 40 beads were imaged for each sample, with images containing out-of-focus beads or bead aggregates being excluded. For the SPR assay, the data collected were generally single measurements, except for two conditions (10 nM, *n* = 2; 25 nM, *n* = 3). Each SPR sensor was used to run two samples across two separate channels, leading to a total of 14 measurements. However, one measurement was excluded due to the loss of a stable signal. The measurements were carried out over two sessions, and one condition (25 nM TcdA) was repeated in both sessions as a control.

### Reporting summary

Further information on research design is available in the Nature Portfolio Reporting Summary linked to this article.

### Supplementary information

Supplementary Information

Reporting Summary

## Data Availability

Data is available in the manuscript and/or supporting information. Amber topology file and final PDB file of each MD simulation run of SA1-SA7, raw data files to reproduce plots are available at: https://github.com/CarolHall-NCSU-CBE/8-mer-peptides-TcdA.

## Code availability

The PepBD code and the matlab code for image processing in the bead-assay is available at: https://github.com/CarolHall-NCSU-CBE/8-mer-peptides-TcdA.
