# Supplementary material for: Design of 8-mer peptides that block Clostridioides difficile toxin A in intestinal cells
Source: Commun Biol. 2023 Aug 26;6:878. doi: 10.1038/s42003-023-05242-x (PMC10460389; doi:10.1038/s42003-023-05242-x)
Supplement: Supplementary file 1 — Supplementary Information [file 42003_2023_5242_MOESM1_ESM.pdf]

# Supporting Information for Publication

# Design of 8-mer Peptides that Block *Clostridioides difficile* Toxin A in Intestinal Cells

Sudeep Sarma[1†], Carly M. Catella[1†], Ellyce T. San Pedro[2], Xingqing Xiao[1], Deniz Durmusoglu[1], Stefano Menegatti[1,3], Nathan Crook[1], Scott T. Magness[2], Carol K. Hall[1*]

1. Department of Chemical Engineering, North Carolina State University, Raleigh NC 27695-7905, USA
2. Department of Medicine, University of North Carolina at Chapel Hill, NC 27514, United States
3. Biomanufacturing Training and Education Center (BTEC), North Carolina State University, Raleigh, NC 27695, United States

[†]These authors contributed equally to this manuscript.

*To whom correspondence should be addressed: hall@ncsu.edu.

**Supplementary Table 1.** Computationally designed 10-mer peptides reported in our previous work[1] that neutralized toxin A in jejunum cells and reference peptide RP[2] with their corresponding $\Delta\Gamma_{score}$ and $\Delta G_{binding}$ values.

| **Peptides** | **Sequences** | $\boldsymbol{\Gamma_{score}}$ | $\boldsymbol{\Delta G_{binding}\left(\frac{kcal}{mol}\right)}$ |
|---|---|---|---|
| RP | EGWHAHTGGG | -37.97 | -8.23 |
| NPA | DYWFQRHGHR | -41.54 | -12.81 |
| NPB | GMFWQHRRHD | -40.60 | -11.35 |
| NPC | DGWIQHYKHR | -39.36 | -6.15 |

## Supplementary Note 1. Amino acid seqences of residues 509-526 on TcdA GTD and TcdB GTD

*"509E, 510Q, 511E, 512I, 513N, 514S, 515L, 516W, 517S, 518F, 519D, 520Q, 521A, 522S, 523A, 524K, 525W, 526Q"*

**Supplementary Table 2.** Classification of the 20 natural amino acids into six residue types according to their hydrophobicity, polarity, size and charge.

| **Residue type** | **Amino Acid** |
|---|---|
| Hydrophobic | Leu, Val, Ile, Met, Phe, Tyr, Trp |
| Negatively charged | Glu, Asp |
| Positively charged | Arg, Lys |
| Hydrophilic | Ser, Thr, Asn, Gln, His |
| Other | Ala, Cys, Pro |
| Glycine | Gly |

**Supplementary Note 2. *In-Silico* Screening of TcdA GTD Binding Peptides (Case 2 and Case 3)**

The lowest scoring peptides obtained from Cases 2 and 3 are SA3 and SA6. Figure 1A and 1B show examples of the plot of the Score ($\Gamma_{score}$) and the RMSD profile v/s the number of sequence and conformation change moves performed in PepBD for Cases 2 and 3. The SA3:TcdA GTD catalytic domain structure obtained from the PepBD algorithm is shown in Figure 1C. Peptide SA3 has a $\Gamma_{score}$ = -43.21, obtained at the 9401$^{th}$ evolution step. Likewise, SA6 has a $\Gamma_{score}$ = -44.37 which is obtained at the 7415$^{th}$ step of the evolution process. The structure of SA6 complexed with the TcA GTD catalytic domain is shown in Figure 1D.

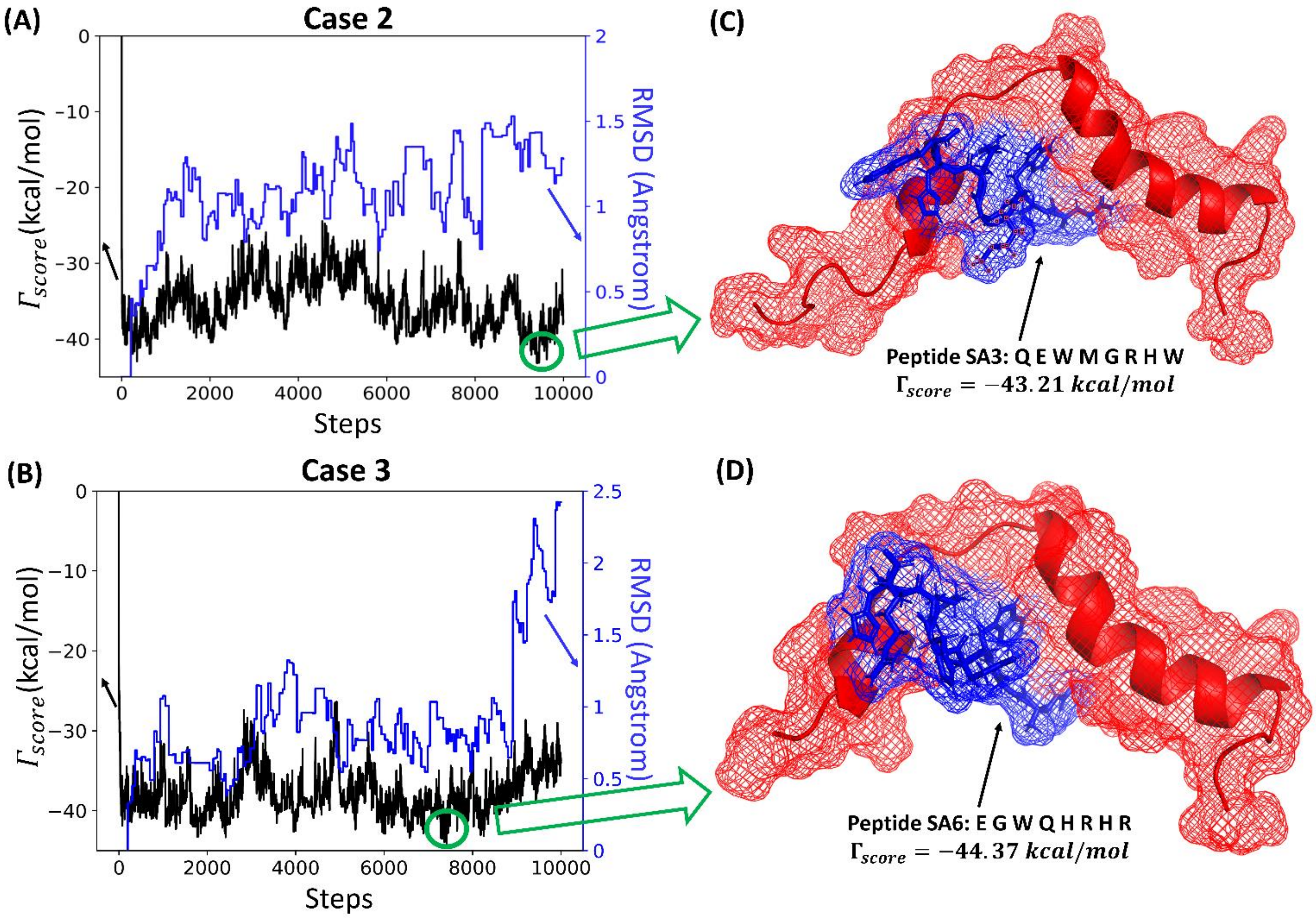


**Supplementary Figure 1.** The score/RMSD vs the number of sequence and conformation steps for **(A)** Case 1 and **(B)** Case 2 with distinct initial random seeds results and their corresponding top peptides **(C)** SA3: QEWMGRHW and **(D)** SA6: EGWQHRHR obtained from PepBD.

**Supplementary Table 3.** Comprehensive list of peptide sequences obtained from PepBD, with their corresponding Case, random seed, $\Gamma_{score}$, as well as if they were evaluated in MD simulations.

| **Peptide Sequence** | **Random Seed** | **Step Number** | $\boldsymbol{\Gamma_{score}} \left(\frac{\boldsymbol{kcal}}{\boldsymbol{mol}}\right)$ | $\Delta \boldsymbol{G_{binding}} \left(\frac{\boldsymbol{kcal}}{\boldsymbol{mol}}\right)$ | **Experimental Testing (Y/N)?** |
|---|---|---|---|---|---|
| Case 1 | | | | | |
| EWQWRRHW | 1 | 7709 | -47.03 | -11.07 | N |
| EFWWRRHN (SA1) | 1 | 459 | -44.59 | -15.94 | Y |
| NEWMRRHW | 2 | 9079 | -50.64 | -2.16 | N |
| QDWMRRHW (SA2) | 2 | 9029 | -50.02 | -13.19 | Y |
| MFWEHRHR (SA4) | 3 | 3468 | -46.74 | -11.01 | Y |
| Case 2 | | | | | |
| EFWMGRHH (SA5) | 1 | 2357 | -42.83 | -6.16 | Y |
| HEWWWRHG | 2 | 1369 | -43.37 | -15.04 | N |
| QEWMGRHW (SA3) | 3 | 9401 | -43.21 | -11.76 | Y |
| Case 3 | | | | | |
| EQWGRRHN | 1 | 9641 | -43.01 | -13.09 | N |
| HEWGRRHN (SA7) | 2 | 3641 | -44.90 | -9.56 | Y |
| EGWQHRHR (SA6) | 3 | 7415 | -44.37 | -12.54 | Y |

**Supplementary Note 3. 500 ns simulations of select peptide:protein complexes**

Since conformational changes can take place over long time scales, we selected SA1:TcdA GTD, SA2:TcdA GTD, SA4:TcdA GTD and SA5:TcdA GTD complexes for performing 500 ns MD simulation each. We calculated the RMSD (root mean squared deviation) with the starting input structure of the peptide:TcdA GTD as the reference structure, over the course of the simulation (Figure 2). The RMSD was recorded every 1 ns and was mass-weighted on the backbone atoms. To determine if there are any significant conformational changes over the course of the simulation, we performed an unpaired t-test on the 96-100 ns time frame and the 101-500 ns time frame (Note that the 96-100 ns timeframe was selected to evaluate the $\Delta G_{binding}$). The unpaired *t-test* confirmed that the difference in the RMSD of the peptide:protein complex over the 95-100 ns time frame and 101-500 ns timeframe is not statistically significant (with 95% confidence), hence, confirming there were no siginificant conformational changes on the peptide:protein complexes over long time scales. The mean and the standard deviation of the RMSD for the 96-100 ns and 101-500 ns timeframes are reported in Table 4.

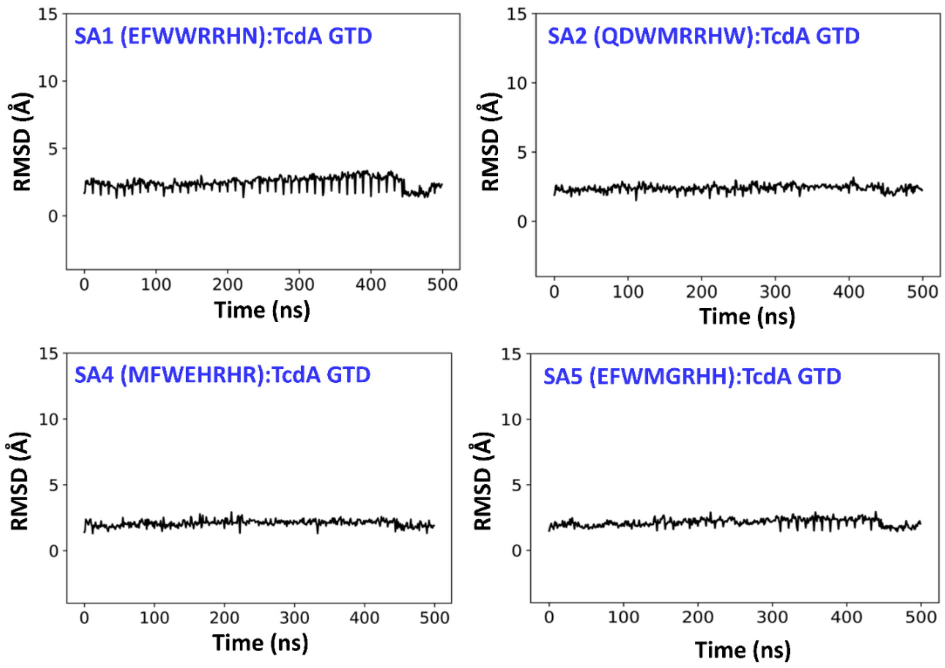


**Supplementary Figure 2.** RMSD (root mean squared deviation) caclulation of SA1:TcdA GTD, SA2:TcdA GTD, SA4:TcdA GTD and SA5:TcdA GTD complexes over the course of 500 ns MD simulation. The reference structure for the RMSD calculation is the starting input structure for each MD run. Snapshots were generated every 1 ns in the simulation.

**Supplementary Table 4.** The mean and the standard deviation of the RMSD for the 96-100 ns and 101-500 ns timeframes for peptides SA1, SA2, SA4 ans SA5.

| **Peptide bound to TcdA GTD** | **95-100 ns** | | **101-500 ns** | |
|---|---|---|---|---|
| | **Mean** | **Standard deviation** | **Mean** | **Standard deviation** |
| SA1 | 2.41 | 0.12 | 2.43 | 0.44 |
| SA2 | 2.42 | 0.15 | 2.37 | 0.22 |
| SA4 | 2.05 | 0.15 | 2.06 | 0.23 |
| SA5 | 1.91 | 0.08 | 2.11 | 0.28 |

We also measured the % secondary structure assignment of the peptides at the 100th, 200th, 300th, 400th and 500th ns over the course of the 500 ns simulation of the four peptides bound to TcdA GTD. Table 5 below summarizes the secondary structure assignment of the peptides. As is expected from short 8-mer peptides, the peptides mostly remain in "coil" or "turn" configuration and at certain points as "3-10 helix".

**Supplementary Table 5.** Secondary structure assignment of peptides SA1, SA2, SA4 and SA5 over the course of 500 ns simulation

| **Peptide** | **100 ns** | | | **200 ns** | | | **300 ns** | | | **400 ns** | | | **500 ns** | | |
|---|---|---|---|---|---|---|---|---|---|---|---|---|---|---|---|
| | **% Coil** | **% Turn** | **% 3-10 helix** | **% Coil** | **% Turn** | **% 3-10 helix** | **% Coil** | **% Turn** | **% 3-10 helix** | **% Coil** | **% Turn** | **% 3-10 helix** | **% Coil** | **% Turn** | **% 3-10 helix** |
| **SA1** | 25 | 75 | 0 | 50 | 50 | 0 | 25 | 75 | 0 | 25 | 75 | 0 | 25 | 75 | 0 |
| **SA2** | 12.5 | 50 | 37.5 | 0 | 100 | 0 | 0 | 100 | 0 | 0 | 100 | 0 | 12.5 | 50 | 37.5 |
| **SA4** | 50 | 50 | 0 | 25 | 75 | 0 | 25 | 75 | 0 | 25 | 75 | 0 | 50 | 50 | 0 |
| **SA5** | 50 | 50 | 0 | 25 | 75 | 0 | 25 | 75 | 0 | 25 | 75 | 0 | 0 | 100 | 0 |

**Supplementary Note 4. Key amino acid interactions of SA1 with TcdA GTD**

Investigation of the biorecognition mechanism of specific residues on the Toxin A neutralizing peptides will help us understand what the key amino acid interactions are on the peptide:TcdA GTD complexes and also support future efforts to design peptides targeting TcdA. When evaluated experimentally, SA1 neutralized toxin A in both primary-derived human jejunum small intestinal and colon epithelial cells and exhibited a $K_D$ of 56.1 ± 29.8 nM measured by surface plasmon resonance (SPR). Hence, to draw further insights as to which SA1 amino acids play key roles in recognizing specific residues on TcdA GTD, we examine the residue-wise decomposition of the interaction energy between SA1 and the catalytic site of TcdA GTD. In Figure 3C of the *Main Text* we plot the residue-wise decomposition of the interaction energy between SA1 and the catalytic site of TcdA GTD. In Figure 3A we construct an energy panel detailing the pair wise interactions of SA1:TcdA GTD complex. Figure 3B shows the number of contacts that each residue on peptide SA1 forms with the TcdA GTD at the binding site. A contact is defined to occur when the distance between a residue on the peptide and a residue on the receptor is ≤ 4.5 Å.

Figures 3C of the *Main Text*, 3A and 3B reveal that the critical residues on SA1 involved in TcdA GTD binding are Trp3, Trp4, Arg5, Arg6, His7 and Asn8. Thus, the four amino acids: tryptophan, arginine, histidine, and asparagine on SA1 play a key role in binding to the TcdA GTD. Phe2 also contributes significantly to the interaction energy but has a slightly lower contribution than the residues mentioned above. It is also worth noting that peptide SA1 contains three aromatic residues (Phe2, Trp3, Trp4), which reflects the importance of aromatic side chains for peptide:TcdA GTD binding. Trp2 and Trp3 both form strong polar- π interaction with Asn513 while cationic Arg5 forms an ionic bond with anionic Glu509. Arg6 forms a cation-π interaction with Trp516 and interacts via coulombic forces with Asn513. His7 and Asn8 form strong polar bonds with Glu509 and Asn330 respectively. The “Number of Contacts” plot also reveals that Trp3, Arg5 and Arg6 form the most contacts with the TcdA GTD at the catalytic domain, further highlighting the importance of tryptophan and arginine in TcdA GTD binding. Glu1 on SA1 does not contribute significantly to the interaction energy but might be necessary to maintain the conformational stability of the peptide, as it can form strong anion-π interactions with neighboring Phe2, Trp3 and Trp4 on the peptide chain. It appears that the 3 three aromatic residues (Phe2, Trp3 and Trp4) also interact with each other on the peptide via π-π interactions and maintain the conformational stability of the peptide.

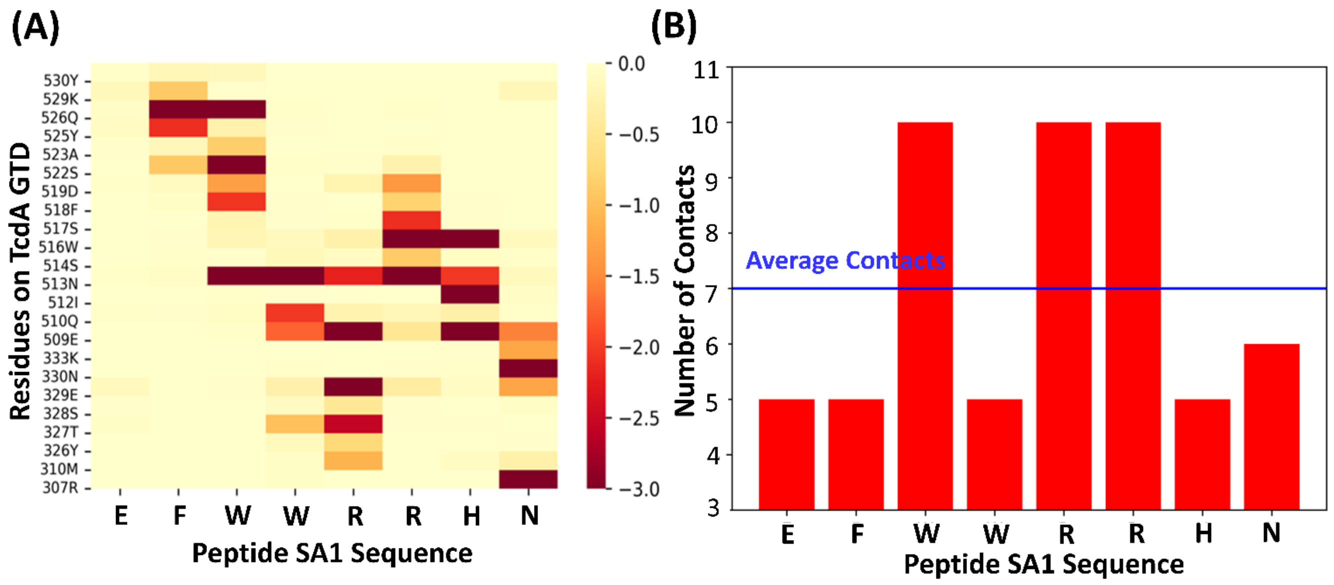


**Supplementary Figure 3. (A)** Energy panel detailing the pair-wise interactions in the SA1:TcdA GTD complex. **(B)** Plot showing the Number of Contacts that each residue forms at the binding interface of the TcdA GTD.

**Supplementary Note 5. Amino acid sequence signatures in *C. diff.* Tcd A GTD binding *in-silico* peptides obtained from PepBD.**

Identifying patterns in the peptide sequences suggested via PepBD gives us an opportunity to obtain signature amino acid sequences that can uniquely bind TcdA GTD with high affinity and specificity. Such patterns offer clues as to why specific amino acids and amino acid types are preferred at certain positions along the peptide chain. PepBD generated ~15000 distinct sequences for Case 1 and ~17000 distinct sequences for Case 2. In Figure 4A, 4B and 4C, we show the sequence homology constructed using Weblogo of the top 1% of the lowest scoring distinct peptide sequences obtained from Case 1, Case 2, and Cases 1 and 2 combined, respectively. In Figure 4D, 4E and 4F, we plot the occupation % by residue type (hydrophobic, polar, positive, negative, glycine) for each site on the peptide chain for Case 1, Case 2, and Cases 1 and 2 combined, respectively. Occupation % by residue type for a site is defined as: $\frac{Number\ of\ residues\ of\ a\ residue\ type}{Total\ number\ of\ residues\ of\ all\ residue\ types\ in\ Top\ 1\%\ of\ the\ lowest\ scoring\ peptides} \times 100$. We also plot the occupation % for the 3 most preferred residues at each site on the peptide chain for Case 1 (Figure 4G), Case 2 (Figure 4H) and Cases 1 and 2 combined (Figure 4I). Similary, Occuptation % of a residue for a site is defined as: $\frac{Number\ of\ times\ a\ residue\ occurs}{Total\ number\ of\ residues\ in\ Top\ 1\%\ of\ the\ lowest\ scoring\ peptides} \times 100$. From Figure 4 it is clear that site 3 on the peptide chain prefers Trp (W), an aromatic hydrophobic amino acid that has an occupation percentage of ~90% for Case 1 and ~100% for Case 2. Trp (W) at site 3 recruits the amino acids on TcdA GTD via both van der Waals and electrostatic interactions. The other two clear favorites are Arg (R) at site 6 and His (H) at site 7. In Case 1, where we specify that the peptide must contain two cationic residues, Arg (R) is preferred over Lys (K) for the 2$^{nd}$ cationic residue on the peptide chain; it occupies either site 5 or 8. In both cases, where we specify that the peptide contain one anionic amino acid, Glu (E) is preferred over Asp (D). Anionic Glu (E) prefers sites 1, 2 or 4 in Case 1 and sites 1, 2 or 8 in Case 2. In Case 2, glycine prefers sites 2, 4, 5 or 8. From the *in-silico* peptide sequence data available, computational binding studies and experimental testing, we can predict amino acid sequence signatures for 8-mer peptides that can bind to *C. diff.* TcdA GTD. The consensus amino acid signature that emerges from Case 1 and Case 2 is given in Table 6.

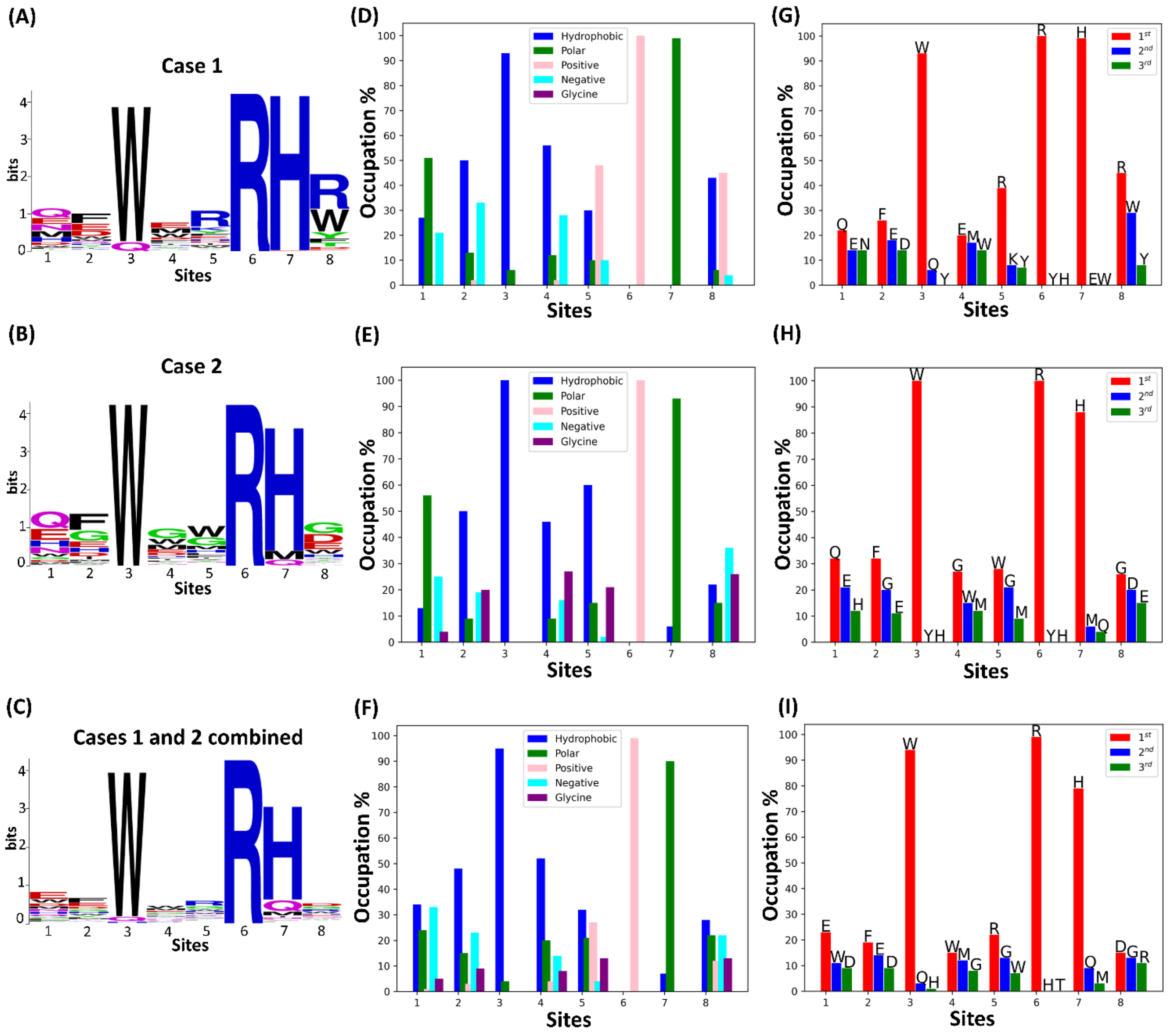


**Supplementary Figure 4.** Identification of amino acid sequence pattern and signature for the top 1% of the lowest scoring distinct peptides obtained from the PepBD algorithm for each case. (A), (B) and (C): Sequence homology of the identified peptides constructed with Weblogo for Cases 1, 2, and 1 and 2 combined, respectively. (D), (E) and (F): Occupation % by residue type at each site along the peptide chain for Cases 1, 2, and 1 and 2 combined, respectively. (G), (H) and (I): Top three amino acids at each site along the peptide chain for Cases 1, 2, and 1 and 2 combined, respectively.

**Supplementary Table 6.** Amino acid signature sequences derived from residue composition of top 1% of the lowest scoring peptides identified by Case 1 and Case 2.

| | **Sites** | | | | | | | |
|---|---|---|---|---|---|---|---|---|
| **Case** | **1** | **2** | **3** | **4** | **5** | **6** | **7** | **8** |
| 1 | Neg/Polar/Pho | Pho/Neg | W | Pho/Neg | Pos/Pho | R | H | Pho/R |
| 2 | Neg/Polar | Pho/Neg/Gly | W | Pho/Gly | Pho/Gly | R | H | X* |

*Here, X can be an amino acid of any residue type

Here, Neg: Negative, Pho: Hydrophobic, Gly: Glycine, Pos: Positive

**Supplementary Note 6. Surface Characterization of SPR Sensors via Ellipsometry and Time-of-Flight Secondary Ion Mass Spectrometry**

The surface thickness of the self-assembled monolayer (SAM) and SAM with peptide SA1 were measured using ellipsometry. The model was fit over the wavelength range from 600-1000 nm to minimize the mean squared error. The mixed thiol SAM had a thickness of 3.01 ± 0.148 nm and the SAM with SA1 had a thickness of 3.76 ± 0.148 nm. The surface thickness of the mixed thiol SAM is consistent with prior work using similar alkane-oligo (ethylene glycol) thiols[3-6], indicating tight packing of a single layer over the surface. The increase in thickness of the SAM with SA1, 7.5 Å, is consistent with the maximum diameter of SA1 estimated from the coordinate file of SA1:TcdA simulated complex, 14.6 Å, and the thickness of other short peptides layers grafted on SAMs[3].

Time-of-flight secondary ion mass spectrometry (ToF-SIMS) was used to characterize the surface of the Surface Plasmon Resonance sensors. Complete coverage by the mixed-thiol self-assembled monolayer (SAM) is necessary to prevent non-specific binding of analyte protein to the gold surface. Analysis of the positive ion spectra shows the complete formation of an alkane-oligo (ethylene glycol) (OEG) SAM as expected. Figure 5A shows characteristic OEG peaks on the SAM sensor, $CH_3O^+$ (*m/z* 29), $C_2H_3O^+$ (*m/z* 43), and $C_2H_5O^+$ (*m/z* 45)[6]. On the SAM-SA1 sensor there is a depletion of OEG peaks and an enrichment in N-containing fragments, including $CH_4N^+$ (*m/z* 30), $C_2H_6N^+$ (*m/z* 44), and $C_4H_8N^+$ (*m/z* 70), indicating engraftment of SA1 onto the SAM (Figures 5A and 5B). Additionally, on the SAM-SA1 sensor there is the appearance of amino acid fragments, including glutamic acid (*m/z* 102), arginine (*m/z* 110 and 112), histidine (*m/z* 110 and 121), and tryptophan (*m/z* 130) (Figures 5C and 5D)[3,7]. The presence of these amino acid signatures further confirms the engraftment of SA1 onto the SAM.

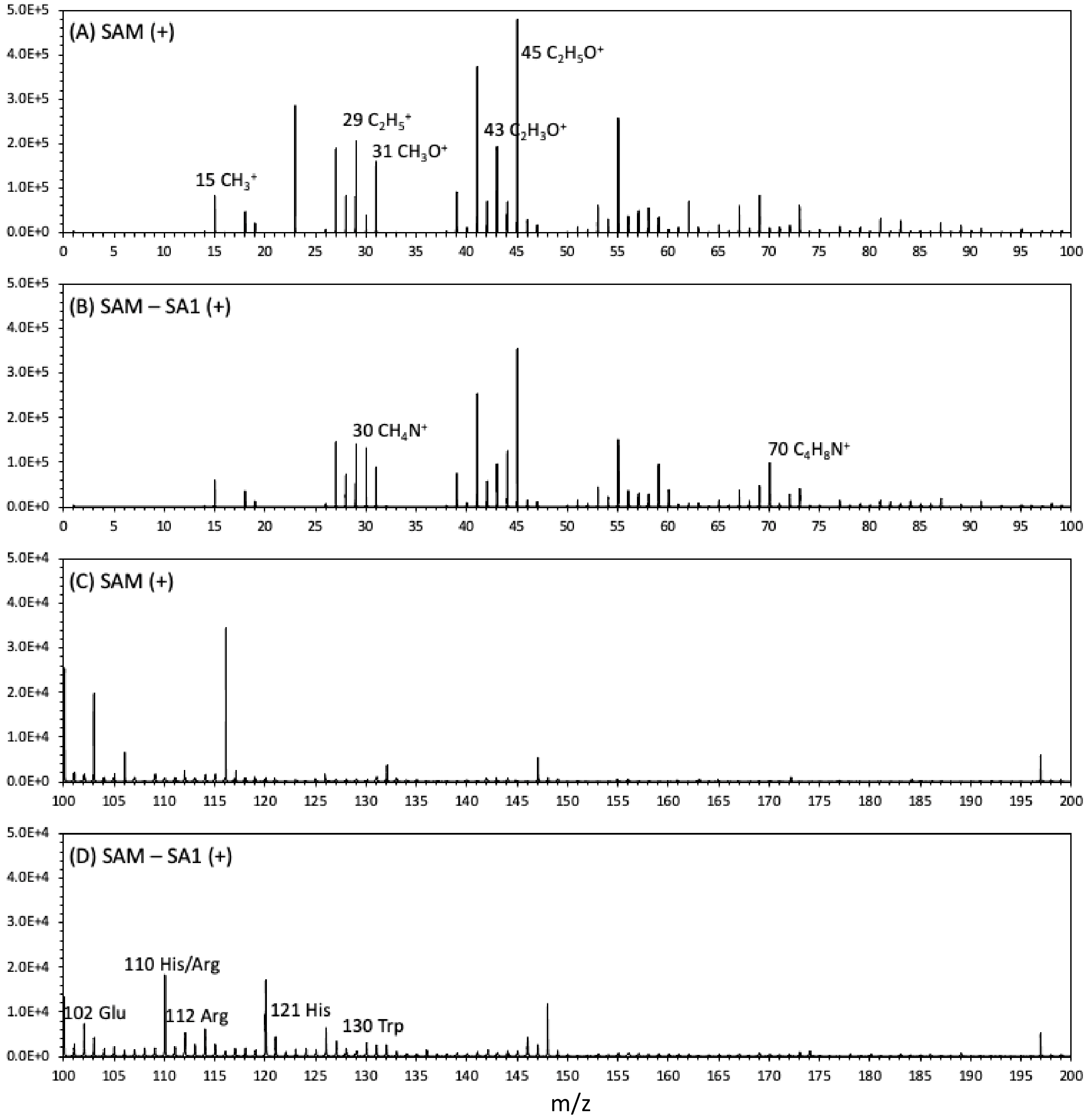


**Supplementary Figure 5.** Positive ToF-SIMS spectra within the range of m/z 0-100 of (A) mixed-thiol self-assembled monolayer (SAM) on gold and (B) SAM-SA1 (SA1 covalently grafted to SAM), and m/z 100-200 of (C) SAM, and (D) SAM-SA1. Key molecular species are labeled.

Analysis of the negative ion spectra further confirms the complete coverage by the SAM and engraftment of SA1. Figure 6A shows the characteristic peaks for SAMs constructed with OEG thiols, $CHO_2^-$ (*m/z* 45), $C_2H_3O_2^-$ (*m/z* 59), $SO_3^-$ (*m/z* 80), and $SO_4^-$ (*m/z* 96)[6]. Similar to the positive ion spectra, the SAM-SA1 sensor showed a depletion of the OEG peaks and an enrichment of N-containing fragments, including, $CN^-$ (*m/z* 26) and $CNO^-$ (*m/z* 42) (Figure 6)[6].

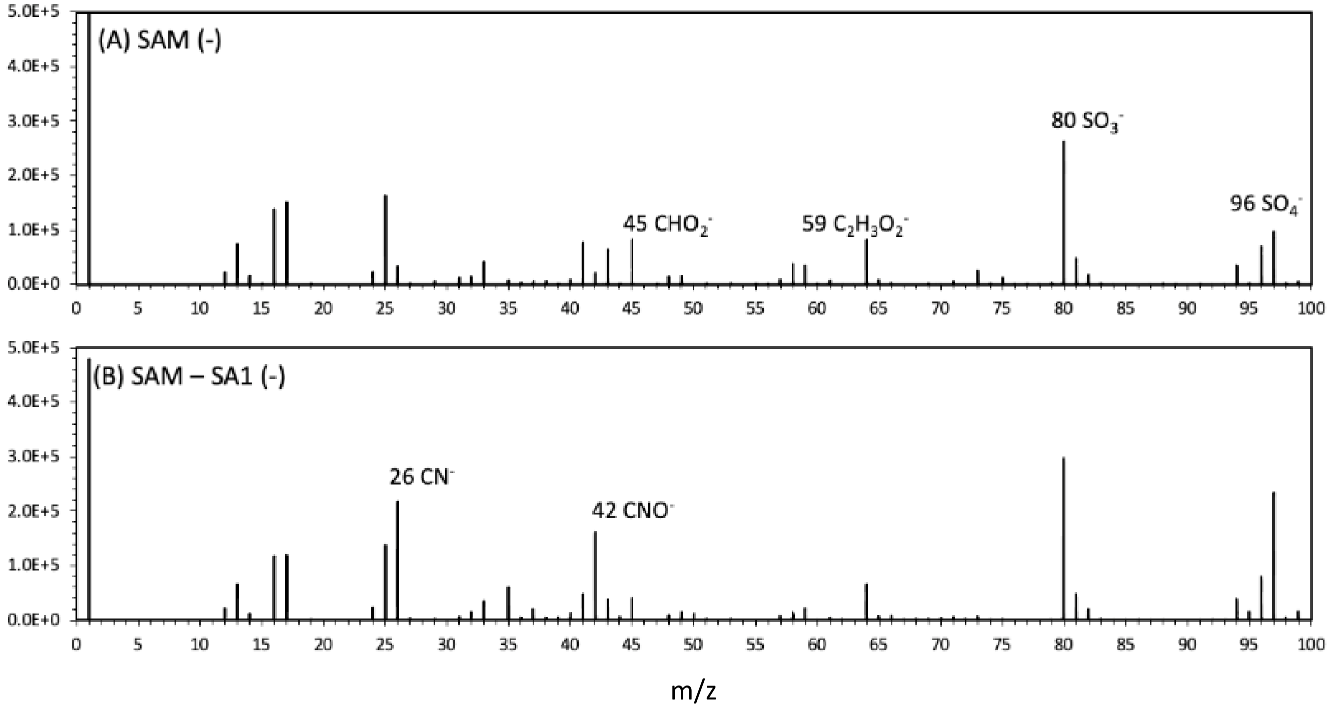


**Supplementary Figure 6.** Negative ToF-SIMS spectra within the range of m/z 0-100 of (A) mixed-thiol self-assembled monolayer (SAM) on gold and (B) SAM-SA1 (SA1 covalently grafted to SAM). Key molecular species are labeled.

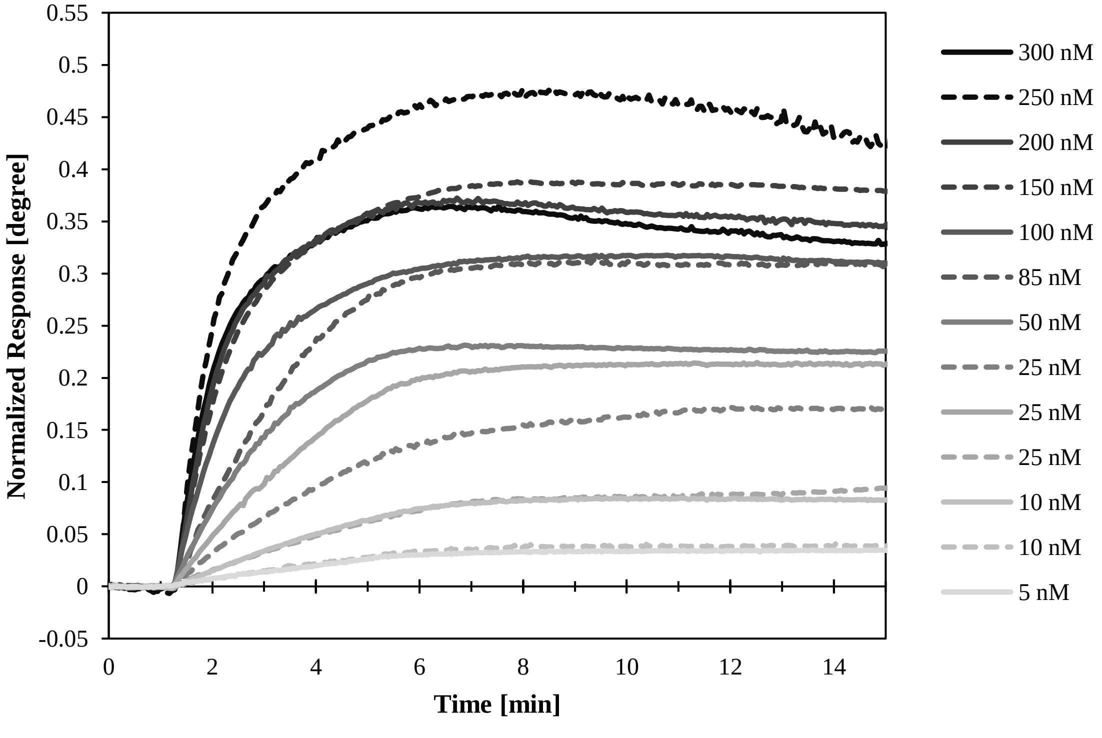


**Supplementary Figure 7.** Raw SPR sensorgrams for TcdA injections (t = 1.2 to 9.5 min) over SA1 functionalized gold sensors. SPR response was normalized with sensor and channel dependent baselines, established for > 5 min prior to injection.

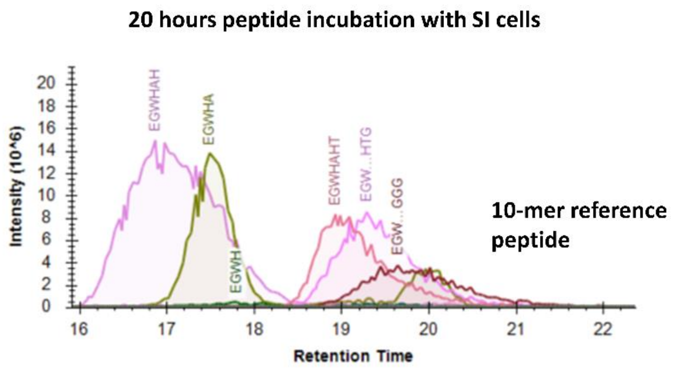


**Supplementary Figure 8.** Mass spectrophotometry on the media containing the 10-mer reference peptide (RP: EGWHAHTGGG) that had been incubated with SI cells.

**Supplementary Note 7. Computational evaluation of the binding affinity of SA1 for TcdB GTD**

The binding affinity of peptide SA1 for the catalytic site on TcdB GTD was evaluated by performing peptide:protein docking, molecular dynamics refinement and $\Delta G_{binding}$ calculation. The protein structure of TcdB GTD corresponding to PDB ID: 2bvl was chosen as the target[8]. The structure was refined by performing a brief 5 ns MD simulation. The coordinate file of peptide SA1 was generated via molecular dynamics simulations in the AMBER 14 suite[9] using the ff14SB forcefield[10,11]. Briefly, a 5ns MD simulation was conducted for the peptide in a simulation box with periodic boundary conditions containing ~3200 water molecules. The resulting peptide was docked *in silico* against the catalytic site (residues 509-526) on the crystal structure of TcdB GTD using the docking software HADDOCK[12]. The resulting poses of SA1:TcdB GTD was clustered based on a fraction of common contacts (FCC), wherein a "cluster" is defined as a collection of at-least 4 structures with > 80% similar contacts. The top binding pose of the first cluster was selected for further MD refinement and $\Delta G_{binding}$ evaluation.

Three three independent simulations of 100ns for the SA1:TcdB GTD complex were carried out. The peptide-receptor complex was solvated in a periodically-truncated octahedral box containing a 12 Å buffer of TIP3P water (~ 36,000 water molecules) surrounding the complex in each direction. Counterions such as Na+ or Cl- were added to neutralize the SA1:TcdB GTD complex prior to running the MD simulations. No additional salt ions were added. The implicit-solvent molecular mechanics/generalized Born surface area (MM/GBSA) approach with the variable internal dielectric constant model was used to evaluate the $\Delta G_{binding}$ from the last 5 ns of the simulation trajectories.

**Supplementary Table 7.** Atomistic molecular dynamics system description

| MD Simulation parameter | Value |
|---|---|
| Simulation box dimesion | 12 Å |
| Total number of residues (6 mer peptide:protein complex) | 545 |
| Total number of residues (7 mer peptide:protein complex) | 546 |
| Total number of residues (8 mer peptide:protein complex) | 547 |
| Total water molecules | ~36,000 |
| Total number of atoms | ~117,000 |

# References


1. X. Xiao, *et al*. In Silico Identification and Experimental Validation of Peptide-Based Inhibitors Targeting Clostridium difficile Toxin A. *ACS Chem. Biol*. **17**, 118-128 (2022).

2. S. J. Abdeen, R. J. Swett, A. L. Feig, Peptide inhibitors targeting clostridium difficile toxins A and B. *ACS Chem. Biol*. **5**, 1097-1103 (2010).

3. N. Islam, F. Shen, P. V. Gurgel, O. J. Rojas, R. G. Carbonell, Dynamic and equilibrium performance of sensors based on short peptide ligands for affinity adsorption of human IgG using surface plasmon resonance. *Biosens. Bioelectron*. **58**, 380-387 (2014).

4. V. Humblot, *et al*., The antibacterial activity of Magainin I immobilized onto mixed thiols Self-Assembled Monolayers. *Biomaterials*. **30**, 3503-3512 (2009).

5. N. Islam, P. V. Gurgel, O. J. Rojas, R. G. Carbonell. Use of a Branched Linker for Enhanced Biosensing Properties in IgG Detection from Mixed Chinese Hamster Ovary Cell Cultures. *Bioconjugate Chem*. **30**, 815-825 (2019).

6. F. Cheng, L. J. Gamble, D. G. Castner. XPS, TOF-SIMS, NEXAFS, and SPR characterization of nitrilotriacetic acid-terminated self-assembled monolayers for controllable immobilization of proteins. *Anal. Chem*. **80**, 2564-2573 (2008).

7. S. Aoyagi, *et al*., Evaluation of Time-of-Flight Secondary Ion Mass Spectrometry Spectra of Peptides by Random Forest with Amino Acid Labels: Results from a Versailles Project on Advanced Materials and Standards Interlaboratory Study. *Anal. Chem*. **93**, 4191-4197 (2021).

8. D. J. Reinart, *et al*., Structural Basis for the Function of Clostridium difficile Toxin B. *J. Mol. Biol*. **351 (5)**, 973-981 (2005).

9. P. K. Weiner, *et al*., AMBER: assisted model building with energy refinement. A general program for modeling molecules and their interactions. *J. Comput. Chem.* **2 (3)**, 287–303 (1981).

10. V. Hornak, *et al*., Comparison of multiple amber force fields and development of improved protein backbone parameters. *Proteins* **65**, 712–725 (2006).

11. W. D. Cornell, *et al*., A second generation force field for the simulation of proteins, nucleic acids, and organic molecules. *J. Am. Chem. Soc.* **117 (19)** 5179–5197 (1995).

12. C. Domingues, *et al*., HADDOCK: a protein-protein docking approach based on biochemical or biophysical information. J. Am. Chem. Soc. **125 (7)** 1731–1737 (2003).
